# Comprehensive analysis of the endoplasmic reticulum stress response in the soybean genome: conserved and plant-specific features

**DOI:** 10.1186/s12864-015-1952-z

**Published:** 2015-10-14

**Authors:** Priscila Alves Silva, José Cleydson F. Silva, Hanna DN Caetano, Joao Paulo B. Machado, Giselle C. Mendes, Pedro AB Reis, Otavio JB Brustolini, Maximiller Dal-Bianco, Elizabeth PB Fontes

**Affiliations:** National Institute of Science and Technology in Plant-Pest Interactions and Departamento de Bioquímica e Biologia Molecular/Bioagro, Universidade Federal de Viçosa, 36570.000 Viçosa, MG Brazil

**Keywords:** *Glycine max*, Unfolded protein response, UPR, Programmed cell death, PCD, ER stress, UPR transducers, Soybean

## Abstract

**Background:**

Despite the relevance of the eukaryotic endoplasmic reticulum (ER)-stress response as an integrator of multiple stress signals into an adaptive response, knowledge about these ER-mediated cytoprotective pathways in soybean (*Glycine max)* is lacking. Here, we searched for genes involved in the highly conserved unfolded protein response (UPR) and ER stress-induced plant-specific cell death signaling pathways in the soybean genome.

**Methods:**

Previously characterized Arabidopsis UPR genes were used as prototypes for the identification of the soybean orthologs and the *in silico* assembly of the UPR in soybean, using eggNOG v4.0 software. Functional studies were also conducted by analyzing the transcriptional activity of soybean UPR transducers.

**Results:**

As a result of this search, we have provided a complete profile of soybean UPR genes with significant predicted protein similarities to *A. thaliana* UPR-associated proteins. Both arms of the plant UPR were further examined functionally, and evidence is presented that the soybean counterparts are true orthologs of previously characterized UPR transducers in Arabidopsis. The bZIP17/bZI28 orthologs (GmbZIP37 and GmbZIP38) and ZIP60 ortholog (GmbZIP68) from soybean have similar structural organizations as their Arabidopsis counterparts, were induced by ER stress and activated an ERSE- and UPRE-containing BiP promoter. Furthermore, the transcript of the putative substrate of GmIREs, GmbZIP68, harbors a canonical site for IRE1 endonuclease activity and was efficiently spliced under ER stress conditions. In a reverse approach, we also examined the Arabidopsis genome for components of a previously characterized ER stress-induced cell death signaling response in soybean. With the exception of GmERD15, which apparently does not possess an Arabidopsis ortholog, the Arabidopsis genome harbors conserved GmNRP, GmNAC81, GmNAC30 and GmVPE sequences that share significant structural and sequence similarities with their soybean counterparts. These results suggest that the NRP/GmNAC81 + GmNAC30/VPE regulatory circuit may transduce cell death signals in plant species other than soybean.

**Conclusions:**

Our *in silico* analyses, along with current and previous functional data, permitted generation of a comprehensive overview of the ER stress response in soybean as a framework for functional prediction of ER stress signaling components and their possible connections with multiple stress responses.

**Electronic supplementary material:**

The online version of this article (doi:10.1186/s12864-015-1952-z) contains supplementary material, which is available to authorized users.

## Background

The endoplasmic reticulum (ER) is a highly dynamic organelle that is involved in major cellular functions, such as protein synthesis, the folding and processing of newly synthesized secretory proteins, protein quality control and the maintenance of Ca2+ homeostasis. Due to the tight regulation of ER homeostasis, this organelle is also involved in the activation of cellular stress responses [1]. The perturbation of ER homeostasis caused by ER stress often promotes the accumulation of unfolded proteins in the lumen, which triggers a cytoprotective signaling pathway referred to as the unfolded protein response (UPR) [2]. In mammalian cells, the UPR operates as a tripartite module, and the ER stress signal is transduced through the ER membrane receptors protein kinase-like ER kinase (PERK), inositol-requiring transmembrane kinase and endonuclease 1α (IRE1) and activation of transcription factor 6 (ATF6) [2].

In plants, the UPR arms, which are mediated by IRE1 homologs and ATF6-related receptors, have been extensively characterized in Arabidopsis and to some extent in rice and maize (for reviews, see [3–5]). Upon disruption of ER homeostasis, plant cells activate at least two branches of the UPR through IRE1-like and ATF6-like transducers, resulting in the up-regulation of ER-resident molecular chaperones and activation of the ER-associated protein degradation system. Recently, a plasma membrane-associated member of the plant-specific NAC domain-containing TF family, AtNAC62, has been demonstrated to undergo cross-talk with ER stress signaling pathways to activate UPR-induced promoters, highlighting a unique aspect of this highly conserved UPR response in plants [6].

Plant IRE1 homologs contain an IRE-like receptor configuration with a stress sensor luminal domain at the N-terminus, a transmembrane segment, and C-terminal kinase and ribonuclease domains. Two IRE1 homologs have been found in Arabidopsis (AtIRE1a and AtIRE1b) and maize and one has been identified in rice (OsIRE1) [3–5]. The substrate for Arabidopsis IRE1 endonuclease activity is the transcript of the ER membrane-associated TF bZIP60 [4, 5]. In response to ER stress, the endonuclease activity of IRE1 mediates the splicing of bZIP60 mRNA to generate an alternatively spliced transcript that lacks transmembrane domain-encoding sequences. This splicing leads to the synthesis of a soluble and functional bZIP transfactor that can be translocated to the nucleus, where it activates ER stress-inducible promoters.

The second branch of the UPR in plants mechanistically resembles the mammalian ATF6-mediated transduction of the ER stress signal. The ATF6 Arabidopsis orthologs include two ER-localized, membrane-tethered TFs, bZIP28 and bZIP17 [3–5]. In the absence of stress, plant BiP is bound to Arabidopsis ATF6-like bZIP28, which remains in the ER membrane [7]. In response to ER stress, BiP dissociates from bZIP28, allowing it to be redirected to the Golgi, where it is proteolytically processed by S1P/S2P and released from the membrane [8]. The released bZIP domain of this transfactor is then translocated to the nucleus, where it acts in concert with the heterotrimeric NF-Y complex to activate UPR genes [9]. In addition to ER stress, bZIP17 is primarily induced by salt stress, a condition that also promotes its regulated movement to the Golgi and S1P/S2P-mediated cleavage, thereby releasing its N-terminal TF domain for translocation to the nucleus, where it acts in concert with bZIP60 to activate salt stress-responsive promoters and a fraction of ER stress-induced promoters [10, 11]. Heat stress induces the expression, S1P/S2P-mediated processing and nuclear translocation of the bZIP28 TF [12]. Maize ZmbZIP17 has been shown to directly link ER stress with ABA signaling [13], and both bZIP28 and bZIP17 connect ER stress and heat stress with BR signaling [14].

The UPR-mediated activation of bZIP60, bZIP17 and bZIP28 promotes the induction of ER-resident molecular chaperones, such as BiP, ERdj, GRP94, CNX, CRT, peptidylprolyl isomerases (PPIases) and thiol disulfide oxidoreductases (PDI and ERp57), through binding to the promoters of the stress-responsive *cis*-regulatory elements UPRE-I and UPRE-II [15]. bZIP60 also transactivates the NAC103 promoter through interaction with a distinct stress-responsive *cis*-regulatory element, UPRE-III [16]. In turn, the NAC103 TF amplifies the UPR signal by further activating several UPR-related chaperones, including CRT1, CNX, and PDI-5 [16]. Downstream components of the UPR also include components of the ERAD machinery, including homologs of EDEMs (MNS4/5), OS9 (EBS6/OS9), Hrd1, Hrd3/Sel1L (EBS5/Hrd3A) and Derlin-1 (Der) [4, 5]. Therefore, under moderate stress conditions, the UPR-mediated induction of ER-resident chaperones and ERAD genes promotes ER quality control processes to re-establish ER homeostasis. However, under prolonged and severe stress, if ER functioning and cell growth cannot be restored, then a cell death program is triggered, presumably to protect the organism from aberrant cells that contain unfolded proteins.

One such plant-specific ER stress-induced cell death response has been recently shown to be mediated by regulated intramembrane proteolysis of the ER membrane-tethered NAC089 TF [17]. In response to ER stress, NAC089 is relocated to the nucleus to control the expression of downstream genes involved in PCD, such as NAC094, MC5 and BAG6. Because the expression of NAC089 is controlled by bZIP28 and bZIP60, during the plant ER stress response, these UPR transducers also elicit pro-death signals, a property that is shared by their mammalian counterparts. A distinct plant-specific ER stress-induced cell death response that integrates an osmotic stress signal into a full PCD response has been reported in soybean and is mediated by the developmental cell death domain (DCD)-containing N-rich proteins DCD/NRP-A and DCD/NRP-B [18]. The expression of DCD/NRP is controlled by the ER and osmotic stress-induced TF GmERD15, which specifically binds to the DCD/NRP promoters to activate the transcription of these genes [19]. Enhanced DCD/NRP accumulation causes the induction of the plant-specific TFs GmNAC81 and GmNAC30, which interact to fully activate expression of the vacuolar processing enzyme (VPE), a plant-specific executioner of programmed cell death (PCD) that displays caspase-1-like activity [20, 21]. Therefore, GmNAC081, GmNAC030 and VPE are involved in a plant-specific regulatory cascade that integrates osmotic stress- and ER stress-induced PCD.

Comprehensive genome-wide evaluations of ER stress-induced changes in gene expression have provided evidence that the UPR operates in a similar fashion in both soybean and Arabidopsis [22]. Nevertheless, genes involved in the ER stress response are poorly characterized in soybean, and except for ER stress-induced NRP-mediated cell death signaling, no other branches of ER stress signaling have been examined at the gene level in this plant. In addition, upstream transducers of the UPR have not been functionally or mechanistically identified in the soybean genome. In this investigation, we conducted a complete survey of upstream, immediate downstream and downstream components of the ER stress response in soybean. Additionally, we examined the possible transducer functions of soybean IRE1 homologs and bZIP28/bZIP17-related receptors. Our *in silico* analyses, along with current and previous functional data, have generated a comprehensive overview of the ER stress response in soybean.

## Results and discussion

The high conservation of the ER stress response in different plant species, such as Arabidopsis and rice, along with the accurate assembly of the soybean genome sequence [23], allowed for the *in silico* identification of components of different branches of the UPR (Table 1) in addition to those of the plant-specific ER stress-induced cell death response (Table 2). Because the plant UPR is transduced as a bipartite module that converges in an adaptive response, we have presented our data in the following groups to facilitate comprehension: UPR transducers/sensors, UPR immediate downstream components and UPR downstream components (Table 1). The corresponding gene copy numbers in the soybean genome are presented in Tables 1 and 2.

**Table 1:** Copy numbers of UPR genes

Arabidopsis designation	Gene copy number in Arabidopsis	Gene copy number in soybean
Transducers/sensors		
bZIP17	1	-
bZIP18	1	-
bZIP17/28	-	2
IRE1A	1	3
IRE1B	1	1
Immediate downstream components		
bZIP60	1	1
NAC103	1	4
S1P	1	2
S2P	1	1
SAR1	2	10
Sec12	3	1
Downstream components		
1) Molecular chaperones/foldases		
BiP	3	4
CRT	3	6
CNX	4	11
PDI	13	22
PPI	1	2
Erdj3	3	8
GRP54	1	2
2) Folding of glycoproteins		
OST	2	4
Glc-I	1	3
Glc-II	3	5
UGGT	1	3
3) ERAD		
MNS3	1	2
MNS4	1	1
MNS5	1	2
EBS6/OS9	1	2
EBS5/HRD3A	1	2
HRD3B	1	Not found
HRD1A-HRD1B	2	2
Der	3	4
PUXUBX2	15	17
UBC32	1	4
UBC33/34	2	2
CDC48	3	10
UFD1	4	6
NPL4	2	2
UDF2	1	2
PNG1	1	2
RAD23	4	7
DSK2	2	4
ER stress/plasma membrane cooperative response		
NAC62	1	3

**Table 2:** Copy numbers of genes involved in ER stress-induced PCD

1) DCD/NRP-mediated cell death response		
Soybean designation	Gene copy number in soybean	Gene copy number in Arabidopsis
GmERD15	2	No description
DCD/NRP-A and B	4 (2 NRP-A and 2 NRP-B)	1
DCD/NRP-C	2	1
NAC81	2	1
NAC30	7	1
VPE	4	2 (alpha-VPE and gamma-VPE)
2) NAC89-mediated cell death signaling		
Arabidopsis designation	Gene copy number in Arabidopsis	Gene copy number in soybean
NAC89	1	2
3) AGB1-mediated cell death signaling		
Arabidopsis designation	Gene copy number in Arabidopsis	Gene copy number in soybean
AGB1	1	4

### Identification of transducers/sensors and immediate downstream components of the UPR

Previously characterized Arabidopsis UPR genes were used as prototypes for the identification of the soybean orthologs and the *in silico* assembly of the UPR in soybean. Using eggNOG v4.0 software, the UPR bZIP transducers bZIP17 and bZIP28 were grouped into the virNOG01396 group, which was comprised of the three genes encoding bZIP17, bZIP28 and bZIP49 (Additional file 1). A search for the bZIP17 and bZIP28 prototypes in eggNOG v4.0 against the Williams 82 v1.1 whole-genome sequence [23] revealed two predicted soybean orthologs (Glyma.03G123200 and Glyma.19G126800, annotated with Phytozome Glyma v.10.1.p, Wm82.a2.v1.1) as the soybean representatives in the virNOG01396 group. A BLASTp search revealed that both of the soybean bZIP gene orthologs were more closely related to bZIP17 (At2G40950). Glyma.03G123200 (GmbZIP38) displayed 60.66 % similarity and 48 % identity to bZIP17 with 96 % protein sequence coverage, and Glyma.19G126800 (GmbZIP37) was 61.32 % similar and 47.68 % identical to bZIP17 with 94 % coverage. The use of the bZIP28 amino acid sequence for comparison resulted in decreases in the similarity and identity of Glyma.03G123200 (GmbZIP38) to 55.99 % and 42.11 %, respectively, with 80 % coverage, whereas Glyma.19G126800 (GmbZIP37) displayed 55.47 % similarity and 41.49 % identity with 81 % coverage (Additional file 1). This level of sequence conservation did not allow us to distinguish between the bZIP17 and bZIP28 soybean orthologs; hence, both GmbZIP37 and GmbZIP38 were assigned as bZIP17/28 orthologs (Table 1).

The immediate downstream components of the bZIP-mediated UPR arm, which are involved in the ER stress-induced mobilization and Golgi-mediated processing of bZIP28 and bZIP17, were also analyzed with eggNOG v4.0. These components included site-1 protease (S1P), a soluble luminal protease, site-2 protease (S2P), a membrane-associated metalloprotease, SAR1, a small GTPase involved in the formation of prebudding complexes for COPII-mediated relocation of cargo from the ER to the Golgi, and SEC12, a COPII vesicle element [5, 24]. The copy numbers of the soybean orthologs are shown in Table 1, and the *e-value* showed a high level of conservation of homologous regions between ortholog pairs (Additional file 1).

The Arabidopsis genome contains three copies of the IRE genes, but only IRE1a (At2G17520) and IRE1b (At5G24360) encode full-length proteins [3–5]. Our *in silico* analysis recovered IRE1a and IRE1b and clustered them into the virNOG09069 group, which encompassed four predicted soybean IRE orthologs. A BLASTp search revealed that the Glyma.01G157800 (GmIRE1a), Glyma.09G197000 (GmIRE1d) and Glyma.11G087200 (GmIRE1c) predicted proteins were the most similar to Arabidopsis IRE1a (80 % similarity and 68 % identity, but different levels of sequence coverage), whereas Glyma.16G111800 (GmIRE1b) was the most similar to IRE1b (60.67 % similarity and 43.14 % identity with 94 % coverage). The *e-value* showed the high conservation of homologous regions among the orthologous proteins.

A striking feature of the soybean genome is the retention of extended blocks of duplicated genes [23]. Approximately 75 % of the 46,430 high-confidence genes predicted to be present in the soybean genome exist as paralogs, and 25 % have reverted to singletons [23]. Phylogenetic analysis of the Arabidopsis and soybean IRE orthologs belonging to the virNOG09069 group (Additional file 1) showed that the four soybean IRE paralogs were clustered in pairs, consistent with duplication events (Additional file 2).

bZIP60 is an immediate downstream component of the IRE arm of the UPR, and its transcript has been shown to serve as an IRE substrate [4, 5, 25]. A search of eggNOG v4.0 for the AtbZIP60 sequence against the soybean genome identified just one soybean ortholog, Glyma.02G161100 (GmbZIP68), which was placed into the euNOG19243 group with significant protein similarity to the AtbZIP60 prototype (Additional file 1). Phylogenetic analysis of soybean and Arabidopsis orthologs of the UPR membrane-tethered bZIP transfactors belonging to the virNOG01396 and virNOG09069 groups confirmed that GmbZIP68 was the most closely related to AtbZIP60 because they were clustered together and separate from the virNOG01396 group of orthologs (Additional file 3). Consistent with a duplication event, soybean GmbZIP37 and GmbZIP38 were clustered together as paralogs, but they were more closely related to AtbZIP17, confirming the eggNOG data (Additional file 1). Predicted protein similarities between soybean and rice bZIP28/17-like genes were also determined (Additional file 3). The orthologous genes in soybean that were the most similar to bZIP17 were also the best matches in rice. The conservation of homologs of these bZIP17-like genes in other species is strongly suggestive of their functional importance and identities.

Recently, an ER stress-induced plant-specific NAC TF, NAC103 (At5g64060), has been shown to be regulated by a functional bZIP60 through the newly identified UPRE-III (TCATCG) on the NAC103 promoter [16]. NAC103 in turn amplifies the UPR signal by up-regulating ER stress-induced promoters, such as CNX and CRT. Using the NAC103 amino acid sequence as a template, we identified four orthologs in the soybean genome (Table 1). eggNOG v4.0 software grouped the NAC103 paralogs ANAC082 and ANAC103 together with the soybean orthologs Glyma.04G213300, Glyma.05G191300, Glyma.08G156500 and Glyma.06G152900 in the virNOG18312 group (Additional file 1). Among them, the Glyma.04G213300 predicted protein, also designated as GmNAC020, displayed the highest sequence similarity to NAC103, whereas Glyma.05G191300 (GmNAC028), Glyma.08G156500 (GmNAC058) and Glyma.06G152900 (GmNAC037) were more similar to ANAC082. As stress-responsive genes, GmNAC020 and GmNAC037 have been shown to be up-regulated by moderate water deficit, whereas GmNAC028 and GmNAC058 are up-regulated by persistent water deficit conditions [26]. The deduced protein sequences of all four NAC103 orthologs from soybean were found to contain a highly conserved NAC domain at the N-terminus that was divided into five NAC subdomains (A–E) of conserved blocks. The presence of an ER stress-responsive element controlled by bZIP60, pUPRE-III, was identified on the GmNAC028 promoter.

### The bZIP- and IRE-mediated arms of the plant UPR are functionally conserved in soybean

The AtbZIP17 and AtbZIP28 TFs are proteolytically activated by inducers of ER stress, such as tunicamycin and DTT, and by adverse environmental conditions, such as heat and salinity [4, 5]. As a consequence, the bZIP domain is released from the membrane and enters into the nucleus, where it regulates the expression of UPR-responsive genes controlled by pERSE (CCAAT-N10-CACG)-, pUPRE (ATTGGTCCACGGTCCATC)-, pUPRE-I (TGACGT-GR)-, pUPRE-II (GATGACGCGTAC)- and/or pUPRE-III (TCATCG or CGATGA)-containing promoters [27, 28].

**Fig. 1:**
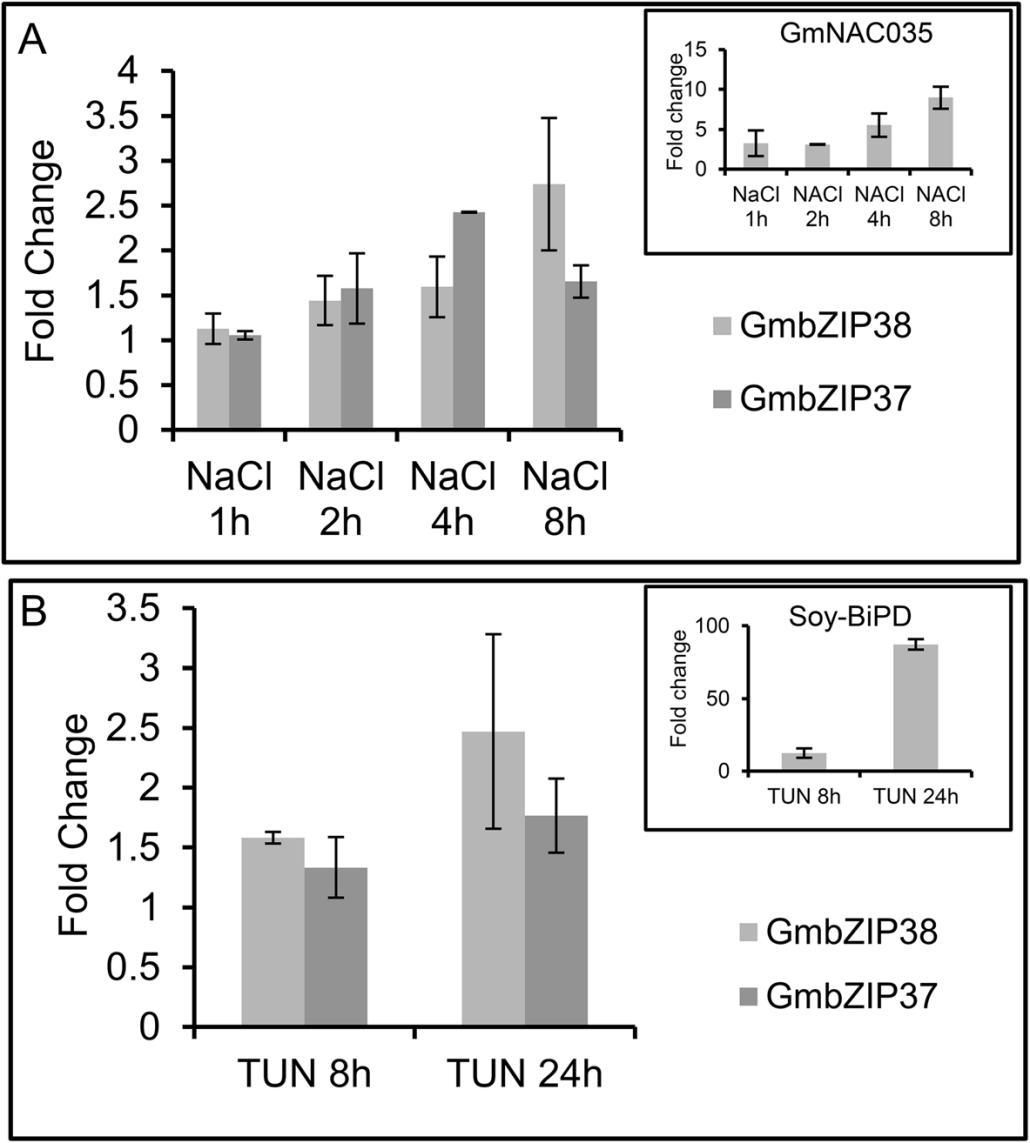
Expression analysis of bZIP38 and bZIP37. Total RNA was isolated from soybean seedlings treated with NaCl (**a**) and tunicamycin (**b**) for the indicated times, and the transcript levels of selected genes (as indicated) were quantified by real-time PCR using gene-specific primers. Gene expression was calculated using the 2^−ΔCT^ method, with helicase as an endogenous control. The error bars indicate 95 % confidence intervals based on t-tests (p < 0.05, *n* = 3)

The functions of soybean GmbZIP38 and GmbZIP37 as bZIP17/28-like UPR transducers were examined using several different approaches. We first analyzed the expression profiles of GmbZIP37 and GmbZIP38 in response to stress conditions known to promote accumulation of unfolded proteins in the ER and to induce AtbZIP17/28 expression, such as ER stress and salt stress. The treatment of soybean seedlings with the salt stress inducer NaCl and the ER stress inducer tunicamycin (which blocks protein glycosylation in this organelle) induced accumulation of the GmbZIP38 (Fig. 1a and b) and GmbZIP37 transcripts with similar kinetics (Fig. 1a and b). Controls for the effectiveness of the salt and ER stress treatments, such as GmNAC035 [29] and BiP (soyBIPD) [30], were also included in the assay (Fig. 1). GmbZIP38 and GmbZIP37 display patterns of expression similar to those of AtbZIP17 and AtbZIP28.

**Fig. 2:**
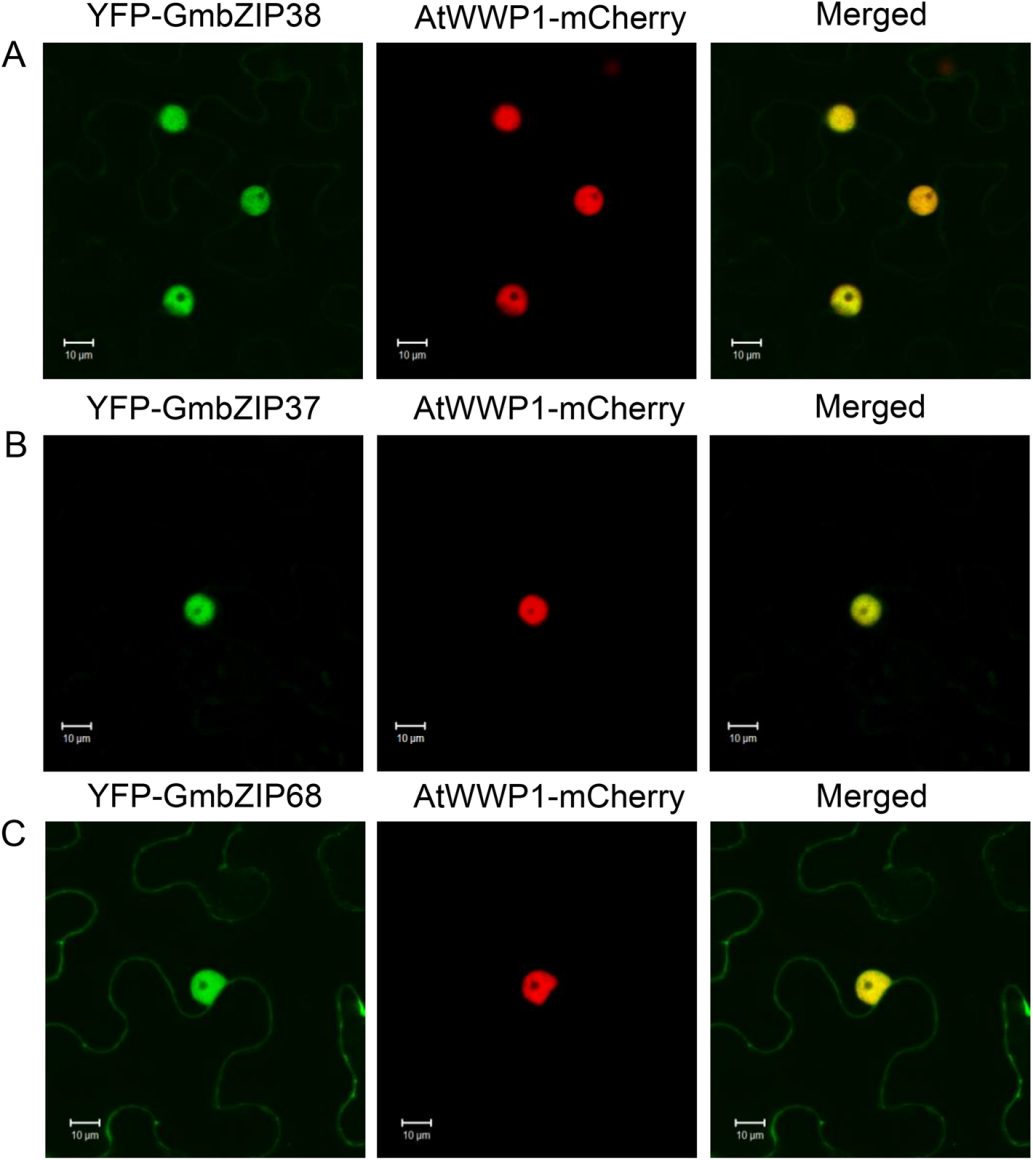
Subcellular localizations of the truncated forms of bZIP38, bZIP37 and bZIP68 fused to YFP. *N. benthamiana* leaves were infiltrated with *Agrobacterium* carrying the indicated DNA constructs. **a** GmZIP38. **b** GMbZIP37. **c** GMbZIP68. The subcellular localizations of the fluorescent fusion proteins were monitored by confocal microscopy at 72 h post-infiltration. The co-localization of the YFP-bZIP fusion proteins with the nuclear marker AtWWP1-mCherry is shown in the merged image. Scale bars = 10 μm

**Fig. 3:**
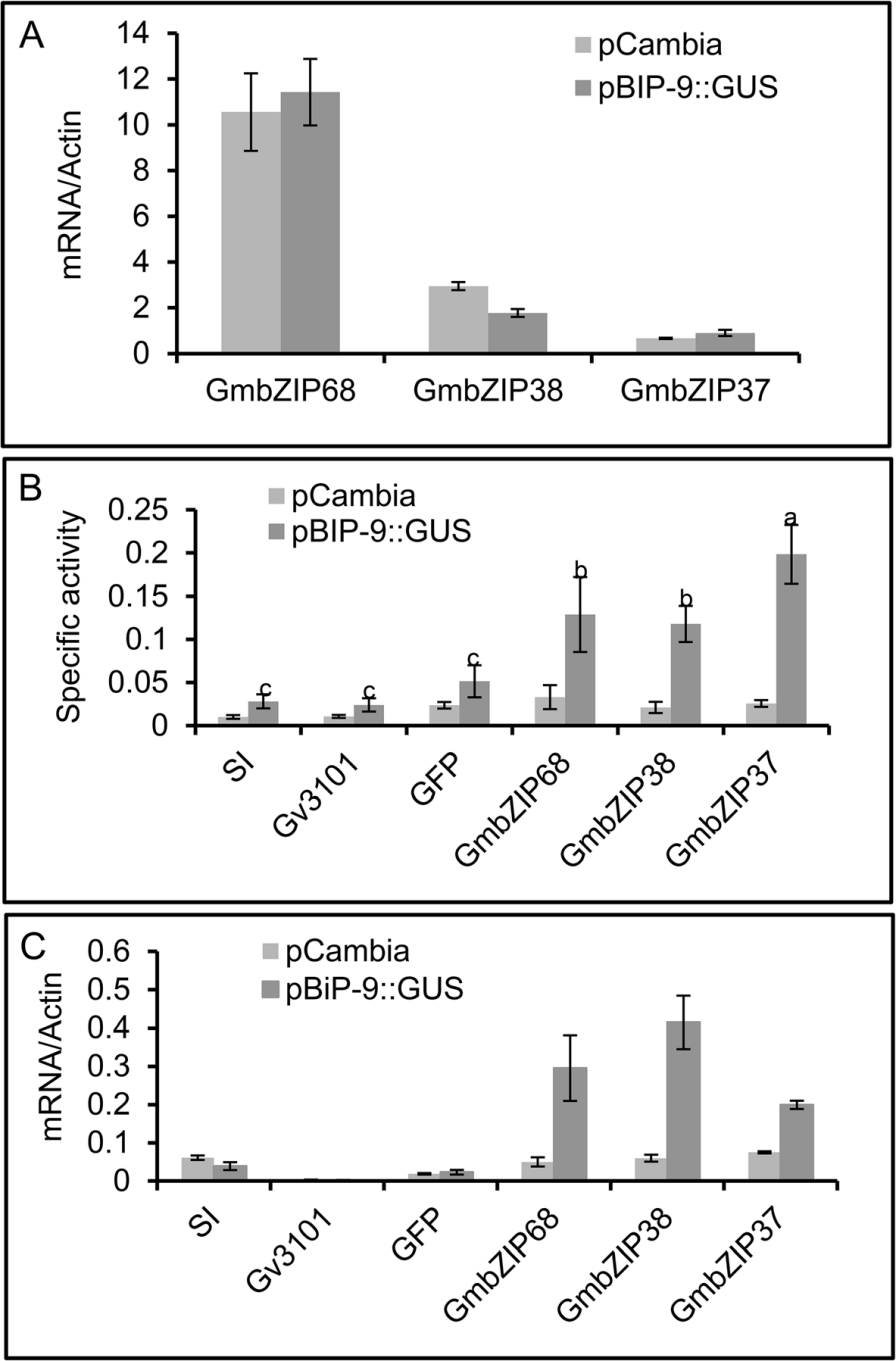
GmbZIP38, GmbZI37 and GmbZIP68 activate a BiP promoter. The leaves of transgenic tobacco lines transformed either with a soyBiPD promoter fused to GUS (pBiP-9::GUS) or an empty pCambia vector (pCambia) were agroinfiltrated with plasmids carrying truncated GmbZIP38 (bZIP38^1–434^), GmbZIP37 (bZIP37^1–406^) and GmbZIP68 (bZIP68^1–209^). **a** Expression of truncated bZIPs in agroinfiltrated leaves. The expression levels of truncated GmbZIP38, GmbZIP37 and GmbZIP68 were analyzed by qRT-PCR at 72 h post-infiltration. Expression levels were calculated using the 2^−ΔCT^ method, with helicase as an endogenous control. The error bars indicate 95 % confidence intervals based on t-tests (p < 0.05, n = 3). **b** Induction of GUS activity in transgenic lines by expression of truncated GmbZIP38, GmbZIP37 and GmbZIP68. Transgenic tobacco leaves (pBiP-9::GUS and pCambia) were infiltrated with *A. tumefaciens* carrying the indicated DNA constructs, and GUS activity was measured at 72 h post-infiltration. Non-inoculated (SI) transgenic lines and those inoculated with either GV3101 or GFP were used as negative controls. **c** GUS transcript accumulation. The expression of GUS was analyzed by qRT-PCR at 72 h post-infiltration. Expression levels were calculated using the 2^−ΔCT^ method, and helicase served as an endogenous control. The error bars indicate 95 % confidence intervals based on t-tests (p < 0.05, n = 3)

To further examine whether GmZIP37 and GmbZIP38 function in the soybean UPR, we searched for bZIP17/28 functional domains in the predicted primary structures of GmbZIP38 and GmbZIP37. Several conserved motifs were found in the GmbZIP38 and GmbZIP37 sequences at corresponding positions in the AtZIP orthologs (Additional file 4 and Additional file 5). These motifs included a bZIP domain at the N-terminal cytosolic-facing region of the predicted proteins, followed by a transmembrane segment and a canonical S1P site (RXXL or RXLX) [4] at the luminal C-terminus (Additional file 5, boxed sequence, RRTL). Based on the mechanistic model of bZIP28 activation and the conserved motifs present in GmZIPs, one may predict that the proteolytic release of GmZIP38 and GmbZIP37 from the ER membrane would render the nuclear-localized bZIP domain functional for regulation of ER stress-induced promoters. To clarify this process, we prepared truncated versions of GmbZIP38 (bZIP38^1–434^) and GmbZIP37 (bZIP37^1–406^) harboring an N-terminal bZIP domain without the transmembrane segment that was fused to YFP, under the control of the 35S promoter (Additional file 4). The truncated YFP-bZIP38^1–434^- and YFP-bZIP37^1–406^ fusion constructs co-localized with the nuclear marker AtWWP1 fused to mCherry in the nuclei of *N. tabacum* epidermal cells when they were transiently co-expressed in leaves (Fig. 2a and b, merged). To provide further evidence that GmbZIP37 and GmbZIP38 are functionally linked to the UPR signaling pathway, we examined whether the truncated bZIP domains directly target ER stress *cis*-regulatory element-containing promoters. We performed *β*-glucuronidase (GUS) transactivation assay using the −2200pbip9-gus tobacco transgenic line stably transformed with a *β*-*GUS* reporter gene under control of the soyBiPD promoter [31]. The soyBiPD promoter harbors repeated ERSEs (with the coordinates −552 to −534, −280 to −260, −219 to −201 and −193 to −175) and a UPRE-I (with the coordinates −185 to −175), which have been previously shown to function as ER stress-responsive elements [31]. We also assessed a control transgenic line stably transformed with a promoterless GUS gene (pCambia empty vector). Accumulation of YFP-bZIP38^1–434^ and YFP-bZIP37^1–406^ transcripts in agroinfiltrated −2200pbip9-gus transgenic leaves and in pCambia control leaves was confirmed by qRT-PCR (Fig. 3a). The effects of promoter transactivation were assessed by measuring β-galactosidase activity (Fig. 3b), as well as by quantifying reporter GUS transcript accumulation (Fig. 3c). The bZIP domains bZIP38^1–434^ and bZIP37^1–406^ specifically activated the BiP promoter, enhancing GUS activity and inducing GUS transcript accumulation in the 2200pbip9-gus transgenic leaves compared with the pCambia transgenic leaves. The infiltration of untransformed *Agrobacterium* culture (Gv3101) and the expression of GFP alone in −2200pbip9-gus transgenic leaves did not result in targeting of the BiP promoter. Collectively, these results implicate GmbZIP37 and GmbZIP38 as true orthologs of Arabidopsis bZIP28 and bZIP17 and suggest that the bZIP28-mediated arm of the UPR is mechanistically conserved in soybean.

**Fig. 4:**
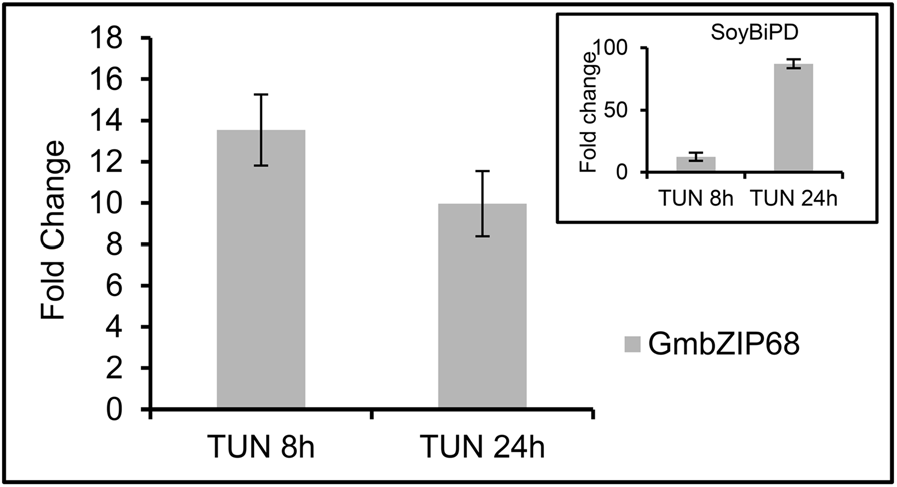
Expression analysis of bZIP68. Total RNA was isolated from soybean seedlings treated with tunicamycin for the indicated time, and the transcript level of the truncated form of bZIP68 was quantified by real-time PCR using gene-specific primers. Gene expression was calculated using the 2^−ΔCT^ method, with helicase as an endogenous control. The error bars indicate 95 % confidence intervals based on t-tests (p < 0.05, n = 3)

**Fig. 5:**
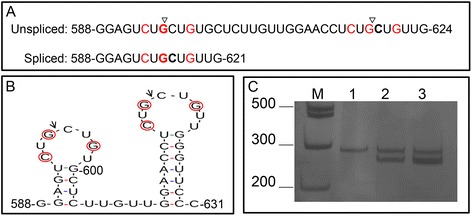
Regulated splicing of bZIP68 caused by ER stress. **a** Predicted spliced sequence of the bZIP68 transcript. The arrows indicate the predicted cleavage sites, and the nucleotides in red are conserved in IRE1 substrates. **b** Loop structure at the predicted splicing site in bZIP68 mRNA. Each of the two loops contains three conserved nucleotides (marked in red) present in IRE1 substrates. **c** ER stress-induced splicing of bZIP68 mRNA. Total RNA was isolated from soybean seedlings that were untreated (lane 1) or treated with tunicamycin for 8 h (lane 2) and 24 h (lane 3) and used as a template for RT-PCR with a combination of primers for spliced and unspliced bZIP68

As the second arm of the plant UPR signaling pathway, upon activation, the dual-functioning protein kinase/ribonuclease IRE1 initiates transduction of the ER stress signal by splicing the bZIP60 transcript. The spliced bZIP60 transcript encodes a truncated version of the protein that lacks a transmembrane domain and C terminus; therefore, the N-terminal bZIP domain is capable of translocating to the nucleus to activate ER stress-responsive genes. In Arabidopsis, bZIP60 is activated and induced by ER stressors and diverse environmental stress conditions that promote accumulation of unfolded proteins in the ER [4, 5]. We found that the bZIP60 ortholog from soybean, GmbZIP68 (Glyma.02G161100), was also induced by the ER stressor tunicamycin (Fig. 4). To examine whether the GmbZIP68 transcript undergoes IRE1-mediated unconventional splicing, we first searched for potential hairpin-like IRE1-specific sites in the GmbZIP68 transcript using RNA structure prediction software (Mfold v2.3). The predicted form of the GmbZIP68 transcript with the lowest free energy is presented in Additional file 6, from which we selected a pair of adjacent hairpin loops, with three conserved bases in each loop (Fig. 5a and b). The selected double-hairpin structure resembles the splicing site in the bZIP60 transcript, which is specifically cleaved by IRE1 at a conserved sequence (CUG↓CUG) in each loop [4]. Based on this bZIP68 twin stem-loop structure with a conserved splice sequence for IRE1 in each loop, we predicted that GmIRE1-mediated alternative splicing of GmZIP68 would remove a 23-nucleotide segment from that site, causing a translational frameshift in the spliced RNA to precisely delete the transmembrane domain, rendering a soluble, functional protein (Additional file 7). To address this possibility, we designed two sets of primers that were each specific for spliced (bZIP68s) or unspliced (bZIP68u) GmZIP68 mRNA (Additional file 7). Primer specificity was confirmed by RT-PCR using cDNA prepared from tunicamycin-treated and untreated soybean seedlings (Additional file 8A). The primers specific for unspliced bZIP68u amplified fragments from both tunicamycin-treated and untreated seedling RNA (lanes 1, 4 and 6), whereas those for spliced bZIP68s amplified a fragment from tunicamycin-treated seedling RNA (lanes 3 and 5) but failed to amplify it from untreated seedling RNA (lane 2), which is consistent with an ER stress-induced splicing event in the target RNA. To detect the removal of the predicted 23b segment of RNA (Fig. 5a and b), the RT-PCR products were separated with a 15 % (w/v) polyacrylamide gel, and RT-PCR was performed using the two sets of primers in the same reaction (Fig. 5c). RNA from untreated soybean seedlings produced an RT-PCR product with a single band on the polyacrylamide gel (lane 1), whereas that extracted from soybean seedlings treated with tunicamycin for 8 h and 24 h generated RT-PCR products with two bands, confirming the ER stress-mediated splicing of GmbZIP68. As a positive control for the ER stress-induced splicing assay, RT-PCR using tunicamycin-treated Arabidopsis RNA with bZIP60u- and bZIP60s-specific primers resulted in the expected double band on a polyacrylamide gel (Additional file 8B).

We also determined the transcription-regulating activity of GmbZIP68 by performing GUS transactivation assays using −2200pbip9-gus tobacco transgenic leaves. We first transiently expressed an N-terminal truncated version of GmbZIP68 (up to amino acid position 209) fused to YFP by agroinfiltrating a 35S::bZIP68^1–209^-YFP construct into *N. benthamiana* leaves and examining its subcellular localization by confocal microscopy (Additional file 4). As expected for a truncated bZIP protein with no transmembrane segment, fluorescence of the YFP-GmbZIP68^1–209^ fusion protein was concentrated in the nuclei of agroinfiltrated *N. benthamiana* leaves (Fig. 2c), co-localizing with the nuclear marker AtWWP1-mCherry (merged). For GUS transactivation assay, *Agrobacterium* carrying a 35S::YFP-GmbZIP68^1–209^ construct or a 35S::GFP negative control was infiltrated into 2200pbip9-gus transgenic leaves and pCambia transgenic leaves. Expression of YFP-GmbZIP68^1–209^, but not that of GFP, activated the BiP promoter, as determined by increases in β-galactosidase activity (Fig. 3b) and transcript levels (Fig. 3c) in the 2200pbip9-gus transgenic leaves compared with the controls. Collectively, these results indicate that GmbZIP68 is a true ortholog of bZIP60 from Arabidopsis and that the IRE-mediated splicing arm of the UPR is functionally conserved in soybean.

Due to the lack of antibodies against GmbZIP37, GmbZIP38 and GmBIP68, we did not monitor the stress-induced accumulation of these UPR receptors at the protein level in the homologous system. Nevertheless, the fusion of GFP to truncated versions of the soybean UPR transducers clearly demonstrated that their bZIP domains accumulated stably in the nuclei of transfected tobacco leaves and functionally activated an ERSE- and UPRE-containing BiP promoter.

### Analysis of UPR downstream components in the soybean genome

To restore ER homeostasis under stress conditions, the plant UPR signaling pathway elicits the up-regulation of stress-specific responses, including increases in protein folding and degradation in the ER. The protein folding capacity of the ER depends on the repertoire of resident molecular chaperones, which has been extensively characterized in Arabidopsis [4, 5]. Therefore, we used the known chaperones from Arabidopsis as the prototypes to identify soybean orthologs though searches of eggNOG. We also searched for typical domains in ER-resident proteins, such as N-terminal peptide signals and C-terminal ER retention signals, as additional criteria to identify soybean orthologs. High degrees of sequence identity/similarity and highly significant e-values were consistently observed between the orthologous pairs of UPR downstream components.

BiP, the most abundant chaperone in the ER, belongs to the heat shock protein 70 kDa (HSP70) family and has been extensively characterized in different plant species, such as tobacco, soybean and Arabidopsis [30–35]. In addition to its molecular chaperone activity, plant BiP also functions in regulating signaling events related to ER stress, and it displays protective functions under distinct stress conditions, including the attenuation of ER stress [18, 36, 37], the promotion of drought tolerance in transgenic soybean (*Glycine max*) and tobacco (*Nicotiana tabacum*) plants [37, 38], the activation of plant innate immunity [39] and the attenuation ER stress- and osmotic stress-induced cell death in soybean [40, 41]. In general, plant BiP is represented by multiple copies (Table 1). A search of eggNOG v4.0 using AtBiP1 as the prototype resulted in the placement of BiP1 and BiP2 from Arabidopsis into the virNOG09258 group together with the previously described soybean BiPs (soyBiPA, soyBiPB/soyBiPD and soyBiPC) and a new additional soybean BiP, gene model Glyma.05G219600 (Additional file 1). AtBiP3 was grouped separately as a virNOG29237 representative, with no closely related homolog in soybean. Phylogenetic analysis of HSP70 members from Arabidopsis and soybean revealed that the BiP proteins were clustered together in a distinct clade, with AtBiP3 placed separate from the others, representing the most distant member of the family (Additional file 9).

Calnexin (CXN) and calreticulin (CRT) represent the major protein folding machinery of the ER, and they specifically bind glycoproteins that carry monoglucosylated N-linked glycans [4]. Calreticulin is a soluble protein in the ER lumen, whereas calnexin is a type 1 membrane protein. In Arabidopsis, three CRT isoforms and two CNX isoforms have been described [42, 43]. A search of eggNOG for the At1G08450 (CRT3) protein sequence against the soybean genome resulted in the clustering of four soybean CRT paralogs into the virNOG02900 group. The other two Arabidopsis CRTs (CRT-1a and CRT-1b) were placed into the virNOG10578 group, together with two soybean CRT orthologs (Additional file 1). The confidence index e-value revealed high conservation of homologous regions between orthologous pairs.

The *A. thaliana* CNX genes were recovered and clustered into five different groups (virNOG06123, virNOG06264, virNOG09352, virNOG13792 and virNOG23307), which included 5 Arabidopsis genes and 11 soybean orthologs (Table 1; Additional file 1). The largest group, virNOG13792, contained the most well-characterized Arabidopsis CNX gene, CNX1 (At5G61790), and four soybean orthologs. All members of the CNX family displayed remarkably conserved primary structures and the conserved domains of ER-resident proteins. As ER-resident molecular chaperones, both calreticulin and calnexin from soybean are induced by ER stressors, such as tunicamycin and AZC.

ER-resident protein disulfide isomerases (PDIs), which are associated with the CNX/CRT system, catalyze disulfide bond formation, which plays relevant roles in the folding and stabilization of tertiary and quaternary protein structures [4, 44]. PDIs are multi-domain proteins that belong to the thioredoxin (TRX) superfamily and hence harbor at least one TRX domain. The Arabidopsis genome encodes 13 PDIs, but only 9 possess known ER retention signals and have been implicated in protein folding [4, 45]. In the soybean genome, 22 PDI paralogs have been previously identified [46]. Our analysis did not result in the selection of the gene model Glyma12g16310 (Phytozome v9.1 as in 70) as a PDI; instead, Glyma.14G152000 (Phytozome v10.1) was included as a new additional PDI paralog in soybean (Additional file 1).

The Arabidopsis PDI paralogs At2G47470, At3G20560 and At4G27080 do not have known ER retention signals [44]. At2G47470 and four highly conserved soybean orthologs (more than 82 % similarity, 70 % identity and 86 % sequence coverage; Additional file 1) were clustered into the virNOG09353 group. The other two PDI paralogs lacking known ER retention signals, At3G20560 and At4G27080, were placed with three soybean orthologs into the virNOG04036 group. The members of this latter group displayed high degrees of sequence conservation with more than 85 % similarity, 71 % identity, 97 % coverage and significant e-values. The soybean PDI orthologs also did not harbor known ER retention signals.

The remaining Arabidopsis PDIs with ER retention signals and 15 soybean orthologs were distributed into seven distinct eggNOG v4.0-generated groups that were significantly conserved (Additional file 1). Phylogenetic analysis results recapitulated the eggNOG data (Additional file 10). PIN1, ERdj and GRP94 orthologs are also represented in the soybean genome by small gene families (Table 1, Additional file 1).

We also identified predicted soybean orthologs involved in glycoprotein folding, such as oligosaccharyltransferase (OST), glucosidase I (Glc-I), glucosidase II (Glc-II) and UDP-glucose:glycoprotein glucosyltransferase (UGGT), using the Arabidopsis homologs as prototypes (Additional file 1) [4, 42]. Remarkable sequence conservation among all orthologs in this category was supported by their significant e-values, and their high levels of similarity and identity suggest that they are functional analogs.

We also examined the components of ERAD in the soybean genome as downstream components of the UPR. The degradation of unfolded proteins by the ERAD system is crucial for the re-establishment of ER homeostasis under stress conditions and involves the following four steps: (i) recognition, (ii) ubiquitination, (iii) retrotranslocation and (iv) protein degradation [4, 42]. In yeast and mammals, the ERAD pathway has been intensively characterized, and this information has been used to identify orthologs in the Arabidopsis genome [42, 47]. The profile of plant ERAD components was extended in this current investigation to include predicted orthologs in soybean. Usa1-like, Cue1-like and OTU1-like proteins were not found in Arabidopsis or soybean. HRD3B–like, PUX6-like, PUX11, PUX12, PUX14 and PUX15 proteins were not detected in the soybean genome. All other ERAD components were represented by at least two related copies in the soybean genome (Table 1). In general, sequence comparison analyses revealed that the predicted ERAD-associated orthologous pairs in Arabidopsis and soybean shared significant amino acid sequence conservation (e-values < 10E-10, sequence similarities and identities of higher than 50 %, with protein sequence coverage of greater than 70 %).

Recently the N-glycan ERAD pathway, which monitors the correct glycosylation of proteins and targets improperly folded glycoproteins for degradation, has been shown to be highly conserved in plants [48–50]. Terminally unfolded glycoproteins are removed from the CNX/CRT folding system through the sequential hydrolysis of two α1,2-mannose residues, as mediated by the ER-resident α1,2-mannosidase MNS3 in Arabidopsis (MNS1 in yeast and mammals and two soybean predicted orthologs) and MNS4/MNS5, which corresponds to homologous to mannosidase 1 (Htm1) in yeast, ER-degradation enhancing a-mannosidase-like protein (EDEM) in mammals and three predicted orthologs in soybean (Additional file 1). The removal of the α1,2-mannose residue exposes a α1,6-mannose residue, which is a targeting signal for the ER-resident lectins EBS6 (Os9 in mammals and Yos9 in yeast) and EBS5/HRD3A (HMG-CoA reductase degradation 3 (Hrd3) in yeast and suppressor of lin-12-like (Sel1L) in mammals) [4, 51]. EBS6 and EBS5 recruit and targets unfolded proteins to the ER membrane-tethered ERAD complex for ubiquitination and retrotranslocation. The Arabidopsis genome has two Hrd3/Sel1L homologs, designated as AtSel1A (EBS5 or HRD3A) and AtSel1B (HRD3B, an apparent pseudogene), and an Os9/Yos9 homolog, AtOs9 (EBS6), whereas in the soybean genome, two representatives in each class with significant sequence identities to their Arabidopsis counterparts were identified (Table 1 and Additional file 1).

The central component of the ERAD complex is a cytosolic-facing ER membrane-associated E3 RING finger-type ubiquitin ligase responsible for the ubiquitination of ERAD substrates and for connection of a series of luminal and cytosolic adapters [4, 5]. The HMG-CoA reductase degradation (Hrd1) protein is specific for ERAD L/M substrates (from membrane or lumen) and degradation of alpha2 (Doa10) ERAD C substrates (from cytosol). The Arabidopsis genome encodes two Hrd1 orthologs (AtHrd1A and AtHrd1B) [51] and two Doa10 orthologs (Doa10A and Doa10B) [4, 52]. Likewise, in the soybean genome, we identified two copies with significant similarities to Hrd1 and Doa10, respectively (Table 1 and Additional file 1). In yeast, Hrd1 E3 ligase-associated proteins include Cue1 (ER anchor protein), UBC6 (membrane-anchored E2), U1-Snp1 associating-1 (Usa1; HERP in mammals) and degradation in the ER (Der1; Derlin, Der1-like protein in mammals). The proteins Cue1 and UBC6 are also contained in the Doa E3 ligase complex. In Arabidopsis, three UBC6 (UBC32, UBC33 and UBC34) homologs are associated with Doa10 [53], whereas in the soybean genome, UBC32 is represented by two copies and UBC34 by two copies. *UBC32* (Ubc6-like E2*)* is induced by salt, drought and ER stress [52]. Neither the Arabidopsis nor the soybean genome encodes the Usa1 or Cue1 gene, but they contain three and four Der1 homologs, respectively (Additional file 1).

Ubiquitinated ERAD substrates are extracted from the ER lumen (ERAD L substrates) or from the ER membrane (M/C substrates) by a trimeric complex of the homohexameric proteins cdc48 (p97 or valosin-containing protein in mammals), Ufd1 and Npl4 (each harboring an ubiquitin-binding domain) [54]. The cdc48/Ufd1/Npl4 complex is recruited by the E3 Hrd1/Doa10 E3 complex through Ubx2 (p97/VCP-interacting membrane protein in mammals, VIMP). The resulting polyubiquitinated ERAD substrates are further processed through antagonistic interactions between ufd2 (U-box-containing E4 multiubiquitination enzyme) and ufd3 (WD40 repeat-containing protein) in addition to Otu1 (deubiquitylating enzyme) and/or by Png1 peptide (cytoplasmic peptide: N-glycanase, PNGase)-mediated deglycosylation [55]. Processed ERAD substrates are directed to the 26S proteasome by Cdc48 and two ubiquitin receptors, Rad23 and Dsk2, for degradation. The Arabidopsis genome encodes three cdc48 homologs (AtCDC48A, AtCDC48B, and AtCDC48C) [56], whereas in the soybean genome, there are 10 predicted proteins with significant similarity to cdc48 (Additional file 1). AtCDC48 is recruited to the ER membrane by UBX domain-containing proteins, which are represented by 15 copies (AtPUXs) in the Arabidopsis genome and interact with AtCDC48A [57]. A total of 17 PUX homologs were identified in the soybean genome (Table 1). While Ufd1, Ufd2, Ufd3, Npl4, Rad23, and Dsk2 are encoded by gene families in Arabidopsis and soybean, PNG is a single-copy gene in the Arabidopsis genome and is represented by two copies in the soybean genome [42]. The high levels of conservation of primary sequences and domain structures among the ERAD components from yeast, mammals and two plant species, along with the findings of functional studies of Arabidopsis (for a review, see [42]) and expression analyses of soybean and Arabidopsis that have been conducted, support the notion that the ERAD system functions in plants in a similar manner as in mammals and yeast.

### Identification of ER stress-induced plasma membrane-associated NAC062 homologs in the soybean genome

Recently, a plasma membrane-tethered member of the NAC family, NAC062, has been shown to integrate UPR signaling through an as-yet-unknown mechanism. ER stress causes the release of the NAC domain from the plasma membrane and its relocation to the nucleus to regulate ER stress-responsive genes [6]. NAC062 expression is controlled by bZIP60. Using eggNOG v4.0 software, GmNAC062 was determined to be a member of the virNOG05505 group, which is comprised of three genes, ANAC062, CBNAC and ANAC091, in addition to three predicted soybean orthologs, GmNAC021, GmNAC036 and GmNAC110 (Table 1; Additional file 1). In addition to displaying significant amino acid sequence similarity, as determined by comparing orthologous pairs, the NAC062 orthologs possess a predicted transmembrane segment and an N-terminal peptide signal that may target them to the plasma membrane. The promoters of the three soybean ortholog genes GmNAC021, GmNAC036 and GmNAC110 harbor a UPR *cis*-regulatory element, pUPRE-III (TCATCG), which is a bZIP60 binding site [16].

### Plant-specific ER stress-induced cell death responses may be conserved in soybean and Arabidopsis

Another plant-specific NAC domain-containing TF, GmNAC089, which is an ER membrane-associated protein, has been shown to play a relevant role in the ER stress response by positively regulating ER stress-induced PCD [17]. ER stress causes relocation of GmNAC089 from the ER membrane to the nucleus, where it induces the expression of PCD-associated genes. A search of eggNOG for GmNAC089 against the soybean genome did not identify any soybean orthologs. Two predicted soybean orthologs, GmNAC103 [58] and an as-yet-unclassified soybean NAC gene (Glyma.12G186900), were identified using BLASTp. This novel full-length NAC predicted protein was recovered from the recently released version of the revised soybean genome, *Glycine max* Wm82.a2.v1, suggesting the existence of two homologous copies of NAC089 in the soybean genome. These soybean orthologs harbor a predicted transmembrane segment and an N-terminal peptide signal that may target them to the ER membrane (Table 2, Additional file 11).

The ER stress- and osmotic stress-induced NRP/DCD-mediated cell death cooperative response, which has been described in soybean, may be the most well-characterized plant-specific ER stress-induced PCD signaling response [18–21]. Consequently, we used soybean genes as prototypes to identify orthologs in Arabidopsis. The GmERD15 (Glyma.14G055200) TF is the most upstream component characterized, and it is induced by osmotic and ER stress to trigger the expression of NRP/DCD genes. A search of eggNOG recovered two paralogs in the soybean genome clustered in the virNOG24368 group and no ortholog in the Arabidopsis genome (Table 2, Additional file 11).

A search of eggNOG for the DCD/NRP-A sequence identified two paralogous copies each of DCD/NRP-A and DCD/NRP-B and a single-copy gene, AtNRP1/At5G42050, in the Arabidopsis genome, with significant amino acid sequence similarities (Additional file 11). These genes were clustered into the virNOG01663 group separately from two soybean paralogs and an Arabidopsis ortholog (AtNRP2/At3G27090) of DCD/NRP-C, which were placed into the virNOG01663 group. Phylogenetic analysis confirmed the separation of DCD/NRP-C from the DCD/NRP-A-DCD/NRP-B cluster (Additional file 12). Although DCD/NRP-A and DCD/NRP-B have redundant and relevant functions in cell death signaling, it remains to be determined whether DCD/NRP-C also functions in the transduction pathway. The high degree of sequence conservation among NRP orthologs from three plant species (rice genome was included in the analysis) may also implicate functional conservation. Consistent with this hypothesis, AtNRP1 and AtNRP2 display similar expression profiles and subcellular localizations as the soybean orthologs [59].

The execution of the cell death program has been proposed to occur through NRP-mediated induction of the GmNAC081-GmNAC030-VPE module [21]. GmNAC081 and its paralog were placed into the virNOG11218 group together with an Arabidopsis ortholog (ANAC036/At2G17040; Additional file 11). In contrast, GmNAC30 was found to be represented by a small multigene family, with seven copies in the soybean genome, which were placed together with the Arabidopsis ortholog ANAC002/ATAF1 (At1G01720) into the virNOG09836 group. Phylogenetic analysis based on the NAC sequences involved in UPR signaling and the ER stress cell death response confirmed the eggNOG data, further supporting the notion that the ER stress-induced NAC orthologs share conserved unique functions in the plant ER-stress response (Additional file 13).

The VPE family has five representatives in the soybean genome [21]. A search of eggNOG for the Glyma.14G092800 sequence against the Arabidopsis and soybean genomes recovered and resulted in the grouping together of four soybean paralogs and two Arabidopsis orthologs (At2G25940/alphaVPE and At4G32940/gammaVPE) into the virNOG04445 group. These data were confirmed by phylogenetic analysis, which revealed that the four soybean VPEs and alphaVPE and gammaVPE from Arabidopsis formed a unique clade that was separated from the fifth soybean VPE, Glyma01g05135 (Additional file 14). The four most closely related soybean VPEs display similar expression profiles during development and in response to stress [41]. The expression profiles and functions of more distantly related VPEs have not been examined. The high conservation of the components of the ER stress NRP-mediated cell death response between soybean and Arabidopsis suggests that this cell death signaling response may be a general ER stress response in plants rather a specific transduction pathway in soybean.

## Conclusions

Despite the relevance of the ER as a key organelle involved in stress adaptive responses, genes involved in the ER stress response in soybean have not been examined to date. Here, we present a complete repertoire of the potential players in the soybean ER stress response, generating a comprehensive panel as a framework for functional predictions.

**Fig. 6:**
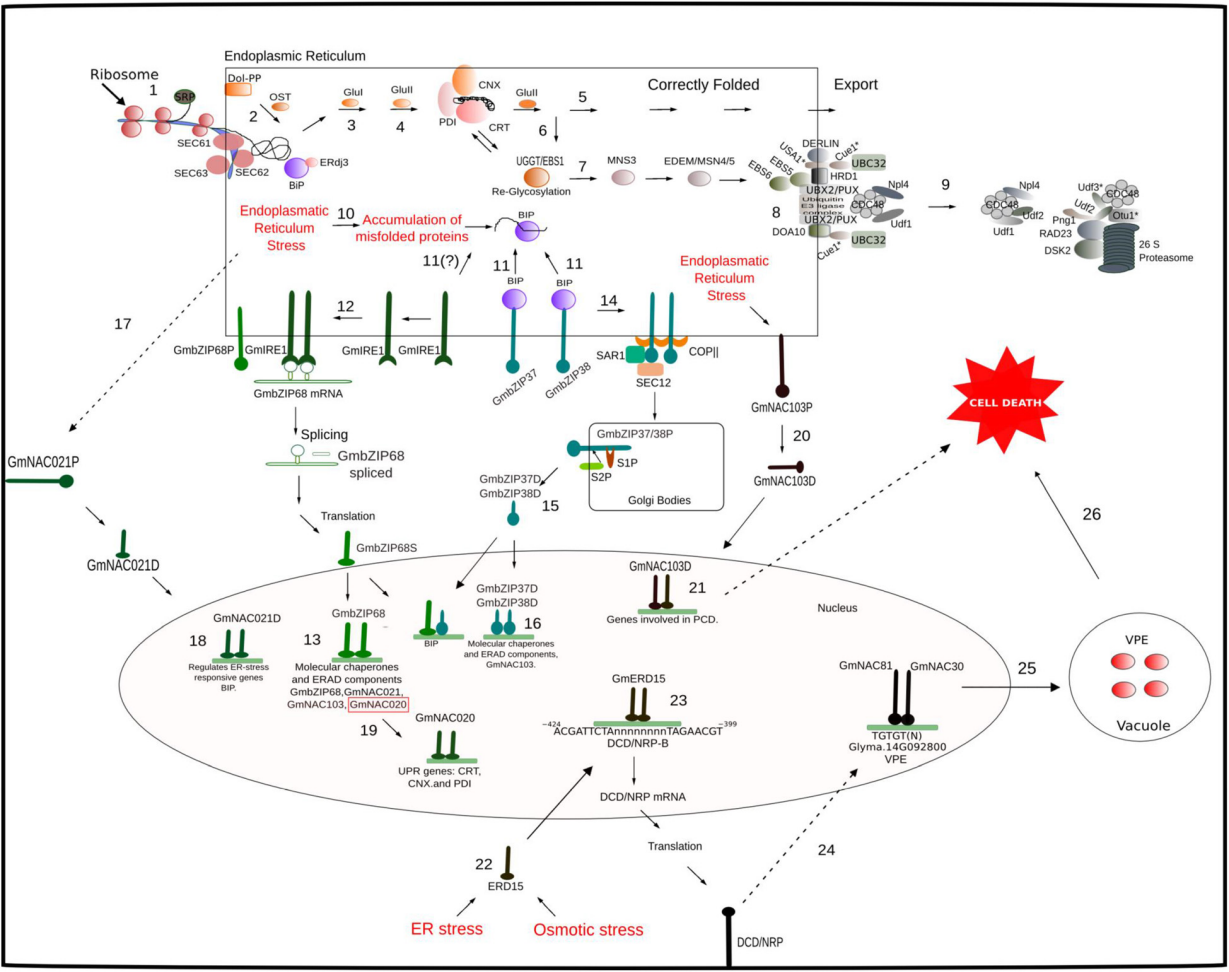
Comprehensive panel of the ER stress response in soybean. To enter the ER lumen, secretory proteins are translated by ER-associated polysomes, and the nascent secretory peptide is co-translationally transported to the ER through the Sec61 translocation complex (1). In the ER, the pre-assembly oligosaccharide core (Glc3Man9GlcNAc2; N-glycan) is transferred (2) from the ER-localized dolichyl pyrophosphate (Dol-PP) to the nascent polypeptide by oligosaccharyltransferase (OST). Processing or trimming of the N-glycan begins in the ER with the sequential removal of the more external glucose residues by glucosidase I (3) and glucosidase II (4). The monoglycosylated glucan-peptide is targeted to the calnexin/calreticulin system containing the protein disulfide isomerase (PDI) accessory protein for proper folding (5). Folded proteins are released from this N-glycan-dependent quality control mechanism through hydrolysis of the third glucose residue by GluII. Properly folded proteins leave the ER. Unfolded proteins may be re-glycosylated by UDP-glucose:glycoprotein glucosyltransferase (UGGT) to re-enter the CNX/CRT-mediated folding cycle (6). The removal of glucose residues and transient re-addition of the innermost glucose during protein folding contribute to the ER retention time of a given glycoprotein. Failure to achieve the proper conformation within a defined period of time is a signal for exclusion of the glycoprotein from the CNX/CRT folding cycle by the sequential removal of two α1,2-mannose residues by MNS3 and MNS4/MNS5 (7). The removal of these residues exposes an α1,6-mannose, which targets the glycoprotein to the ERAD pathway. EBS6 and EBS5 recruit unfolded glycoproteins to redirect them to the membrane-associated ERAD complex for ubiquitination and retrotranslocation to the cytosol, where they are targeted to the proteasome (9). Ubiquitinated ERAD substrates are directed from the ER to the proteasome via the trimeric complex cdc48/Ufd1/Npl4 (9). ER stress induces the accumulation of unfolded proteins in the lumen and activates the UPR pathway (10). BiP-mediated dissociation 0of the UPR transducers GmbZIP37/38 (AtbZIP17/28) (11) allows for the mobilization of these receptors to the Golgi (14), where they are proteolytically cleaved by S1P and S2P (15), releasing the N-terminal bZIP domains as functional TFs that are then translocated to the nucleus (15), where they activate ER stress-responsive promoters (16). In the other arm of the UPR (12), under ER stress, GmIRE1 dimerizes to activate its ribonuclease activity, which promotes unconventional splicing of the GmbZIP68 (AtbZIP60) mRNA, generating an active TF (GmbZIP68S) lacking the transmembrane segment. GmbZIP68S (AtbZIP60S) moves to the nucleus to induce the expression of molecular chaperones, ERAD components, GmbZIP68 (AtbZIP60), GmNAC021 (AtNAC062) and GmNAC103 (AtNAC089). Evidence indicates that GmbZIP37/38 and GmbZIP68S may act in concert as heterodimers to activate ER stress-responsive genes. GmbZIP68S (AtbZIP60S) also induces expression of the TF GmNAC020 (AtNAC103) to further amplify the ER stress response (19). As a plasma membrane component of the ER stress response (17), membrane-tethered GmNAC021 (AtNAC62) also undergoes regulated intramembrane proteolysis (RIP) for release into the nucleus as a positive regulator of ER stress-responsive genes. If the UPR is not capable of restoring ER homeostasis under prolonged and severe stress, then PCD responses are activated for the regulated disposal of abnormal cells. ER stress-induced proteolysis of ER membrane-tethered GmNAC103 (AtNAC089) exemplifies an ER stress-induced plant-specific PCD response (20). RIP-mediated translocation of GmNAC103 to the nucleus allows for the induction of PCD-associated gene expression, promoting DNA fragmentation and an increase in caspase-3/7-like activity. A distinct ER stress-induced PCD response in soybean integrates an osmotic stress signal into a full response (22). The combination of ER stress and osmotic stress fully induces the expression of the TF GmERD15 (22) to activate the expression of the membrane-associated protein DCD/NRP-B (23). Induction of DCD/NRP-B activates a signaling cascade that culminates with the induction of the GmNAC081 and GmNAC030 TFs (24), which form heterodimers to fully transactivate the vacuolar processing enzyme (VPE) promoter (25). VPE exhibits caspase-1-like activity and induces plant-specific PCD, mediated by collapse of the vacuole (26)

As the major result of our research, a complete scenario of the ER stress response in soybean is presented in Fig. 6. An interactive map of this comprehensive panel of the ER stress response is also available at the address http://inctipp.bioagro.ufv.br/upr/. This tool enables the access of detailed information about the protein families in the soybean database by clicking on the representative gene in the panel. In Fig. 6 and in its online version, the normal pathway of secretory proteins as they enter the ER lumen and proceed towards the Golgi is depicted in numbers 1 through 6. Disruption of proper folding results in deviation from this route (number 6) to a protein degradation pathway, shown in numbers 7 through 9. Accumulation of unfolded proteins activates UPR signaling, which functions as a bipartite module. The ATF6-like-mediated arm of the UPR can be followed in numbers 11, 14, 15 and 16, whereas the IRE1-like-mediated arm is presented as route 12. Plant-specific cross-talk between ER stress response pathways and plasma membrane-associated proteins is presented as route 17. We also identified representatives of the plant-specific ER stress-induced cell death response in the soybean genome. In route 20, transduction of an ER stress-induced signal starts with the predicted regulated intramembrane proteolysis of a membrane-tethered NAC domain-containing TF. The mechanism of execution of the cell death program is lacking, with the exception of the observation that the released NAC TF up-regulates cell death-associated genes. The ER stress- and osmotic stress-induced cell death response is initiated in number 22 and culminates with activation of the expression of VPE (number 24), which is an executioner of plant-specific vacuole collapse-mediated PCD. In soybean, similar to other eukaryotic organisms, ER stress triggers the evolutionarily conservative UPR and also accommodates cross-talk with several other adaptive signaling responses, such as osmotic-stress induced cell death and ER stress-induced PCD.

We provided several lines of evidence that the soybean and Arabidopsis ER stress responses operate similarly. First, in addition to the high conservation of the primary structures of the soybean and Arabidopsis putative orthologs, they share common functional and localization domains that may be associated with their shared biochemical activities and subcellular localizations. Second, both arms of the UPR were further examined functionally, and evidence is presented that the soybean counterparts are true orthologs of previously characterized UPR transducers in Arabidopsis. The bZIP17/bZI28 orthologs (GmbZIP37 and GmbZIP38) and ZIP60 ortholog (GmbZIP68) from soybean are induced by ER stress and activate an ERSE- and UPRE-containing BiP promoter. Furthermore, the transcript of the putative substrate of GmIREs, GmbZIP68, harbors a canonical site for IRE1 endonuclease activity and is efficiently spliced under ER stress conditions, generating a truncated version of the protein that lacks the transmembrane segment and includes a second nuclear localization signal. These expression and functional analyses of bZIP38, bZIP37 and bZIP68 support the notion that the bipartite module of the plant UPR is functionally conserved in soybean. Finally, in a reverse approach, we showed that the ER stress-induced DCD/NRP-mediated PCD response, which has been previously characterized in soybean, is also conserved in Arabidopsisand in rice. The components of this ER stress-induced cell death signaling pathway are also induced by other biotic and abiotic signals, such drought, salt and pathogen incompatible interactions [20, 25, 29]. Therefore, activation of the NRP-mediated PCD response is not specific to ER stress; rather, it is a shared branch of general environmental adaptive pathways.

## Methods

### *In silico* identification of unfolded protein response components in soybean

We first searched in the literature for previously described *Arabidopsis thaliana* UPR genes, including those encoding both upstream receptors (ER stress sensors) and downstream components involved in ERAD and the processing and folding of secretory proteins. These Arabidopsis genes were used as reference genes for the identification of UPR components in soybean (Additional file 1, reference list). The soybean genes involved in ER stress-induced NRP-mediated cell death signaling, a plant-specific ER stress-induced cell death response that has been previously described in soybean, were used as prototypes to search for counterparts in the Arabidopsis genome (Additional file 11, reference list). Using eggNOG (Evolutionary Genealogy of Genes: Non-supervised Orthologous Groups) database v.4.0 (http://eggnogdb.embl.de/#/app/home) [60], we identified orthologous plant genes from virNOG (Virideplantae NOG)-predicted groups. Using a locally developed script, Arabidopsis and soybean orthologous genes were extracted from eggNOG v4 database (Additional file 1: Table S1 and Additional file 11: Tables S2). Because eggNOG v4 includes the soybean genome assembly *Glycine max v1.1 *(http://www.phytozome.net/search.php?show=text&method=Org_Gmax), we also used the soybean genome assembly *Glycine max* Wm82.a2.v1 that was recently released by Phytozome v10.1 (http://phytozome.jgi.doe.gov/pz/portal.html#!info?alias=Org_Gmax) to update the annotations of the version v1.1 genes. Therefore, the annotations of the soybean genes were recovered from Phytozome v10.1 (http://phytozome.jgi.doe.gov/).

The group of *A. thaliana* orthologous genes initially recovered from the virNOG dataset were complemented by performing searches of the euNOG and KOG modules implemented in eggNOG v4.0 (Additional file 1 and Additional file 11). The amino acid sequences of orthologous genes from *A. thaliana* and soybean were recovered from TAIR (http://arabidopsis.org/) and Phytozome v10.1 databases, respectively. Pairwise amino acid sequence comparisons between each member of a group were performed using Basic Local Alignment Search Tool (BLAST), with an *e-value* cut off of ≤ 10E-10. This approach led to the identification of soybean orthologs that were more closely related to the Arabidopsis genes based on the criteria of greater identity, similarity and sequence coverage, which were supported by the *e-value* confidence index.

### *In silico* validation of the AtbZIP17, AtbZIP28 and AtbZIP60 orthologous genes from soybean

For the *in silico* functional characterization of AtbZIP17, AtbZIP28, AtbZIP60, AtNAC089 and AtNAC062 orthologs from soybean, we analyzed the presence of protein domains, the putative locations of the orthologous predicted proteins and the topology of transmembrane helices. For the identification of protein domains, we used PFAM database (http://pfam.xfam.org/) and HMMer tools (hmmer.janelia.org), which are both available in SMART v.7.0 web server (http://smart.embl-heidelberg.de/). The transmembrane helix segments were identified using TMHMM software (http://www.cbs.dtu.dk/services/TMHMM/).

### Phylogenetic analyses of the IREs, bZIP17, bZIP28 and bZIP60 genes

Initially, we constructed a dataset that included the IRE genes from soybean (Glyma.01G157800, Glyma.09G197000, Glyma.11G087200, and Glyma.16G111800) and the Arabidopsis orthologous genes (AT2G17520 and AT5G24360), all of which belonged to the virNOG09069 group (Additional file 1). A second dataset contained the soybean bZIP genes (Glyma.03G123200 and Glyma.19G126800, Glyma.02G161100) and the potential orthologous genes from *A. thaliana* (AT2G40950, AT3G10800, AT3G56660, and AT1G42990 and bZIP17, bZIP28, bZIP49 and bZIP60, respectively), all of which belonged to the virNOG01396 and euNOG19243 groups, respectively (Additional file 1). Unrooted phylogenetic trees were constructed using the maximum likelihood method with 10,000 bootstrap replications and the Jones-Talor-Thornton (JTT) amino acid substitution model with MEGA v.6 software. The trees were visualized with Figtree v1.4 software (http://tree.bio.ed.ac.uk/software/figtree/).

### Plasmid construction

The N-terminal cytoplasmic domain of Glyma.02G161100, which spans from bp positions 1 to 627 in the cDNA or from amino acid positions 1 to 209 in the predicted primary structure (Additional file 4C), was isolated from soybean (cv. Conquista) cDNA via PCR using specific primers with appropriate extensions for cloning with Gateway (Life Technologies) (Additional file 15). Similarly, the cytoplasmic domain of glyma03g27865, spanning from bp positions 1 to 1302 or from amino acid positions 1 to 434 (Additional file 4A), and of Glyma.19G126800, spanning from bp positions 1 to 1218 or amino acid positions 1 to 406 (Additional file 4B), were isolated via PCR using gene-specific primers (Additional file 15).

The amplified products were examined by electrophoresis on 1 % (w/v) agarose gels, purified using a Gel Extraction Kit (Qiagen) and inserted by recombination into an entry vector, pDonR207 (Life Technologies). The resulting clones pUFV2325 (Glyma.02G161100-pdonR207), pUFV2506 (glyma03g27865-pdonR207) and pUFV2423 (Glyma.19G126800-pdonR207), contained the fragments of the indicated genes covering the N-terminal domain-encoding region up to the transmembrane segment (Additional file 4). These pDonR207-derived clones were used to transfer the respective inserts to a plant expression vector, pEarleyGate-104, generating pUFV2554 (Glyma.02G161100-pEarleyGate-104), pUFV2555 (glyma03g27865-pEarleyGate-104) and pUFV2556 (Glyma.19G126800-pEarleyGate-104), which contained the respective truncated cDNA fragment fused to the C-terminus of yellow fluorescent protein (YFP*)* under the control of the 35S promoter.

### Plant materials

The tobacco *(N. tabacum* cv. Havana*)* transgenic line BIP-9::GUS has been previously described [31]. The BIP-9::GUS line harbors the promoter (2000 bp of the 5’-flanking region) of the genomic BIP-9 clone (soyBIP cDNA) fused to the reporter gene GUS contained in a plant binary expression vector, pCAMBIA1381z. The pCambia::GUS transgenic line, harboring an empty pCAMBIA1381z vector with a promoterless GUS gene, was used as a negative control [31]. Seeds from BiP9::GUS and pCambia::GUS lines were germinated *in vitro* in Murashige and Skoog (MS) medium supplemented with 25 mg.L^−1^ hygromycin, and they were maintained in a growth chamber at 22 °C under a 16 h light/8 h dark cycle for 16 days. Then, the seedlings were transferred to 40-mL pots containing MS medium and hygromycin. At 35 days post-germination, the plants were transferred to the commercial substrate Tropstrato HT and were maintained in growth chambers at 22 °C under a 16-h photoperiod for 42 days for transactivation GUS assays.

### Transient expression in *Nicotiana benthamiana* leaves and *Nicotiana tabacum* cv. Havana transgenic lines by agroinoculation

The *Agrobacterium tumefaciens* strain GV3101 carrying Glyma.02G161100-pEarleyGate-104 (pUFV2554), glyma03g27865-pEarleyGate-104 (pUFV2555) or Glyma.19G126800-pEarleyGate-104 (pUFV2556) DNA constructs was grown for 12 h and subsequently centrifuged for 5 min at 5,000 x *g*. Pelleted cells were washed with 1 mL of infiltration medium (10 mM MgCl_2_, 10 mM MES, pH 5.6, and 100 μM acetosyringone) and concentrated to an OD_600nm_ = 0.5. *Agrobacterium* infiltration was performed with 3-week-old *N. benthamiana* leaves and 42-day-old BIP-9::GUS and pCambia::GUS transgenic leaves using sterile syringes under manually controlled pressure. After 72 h, infiltrated leaves from *N. benthamiana* were examined by confocal microscopy, and those from *N. tabacum* cv. Havana transgenic lines were used for GUS transactivation assays. *Agrobacterium* transformed with an unrelated DNA construct, At2g41020 – AtWWP1 (pUFV2224), was used as a control for nuclear localization.

### Subcellular localization assay

To examine the subcellular localization of proteins, *N. benthamiana* leaves were agroinoculated with Glyma.02G161100-pEarleyGate-104 (pUFV2554), glyma03g27865-pEarleyGate-104 (pUFV2555) or Glyma.19G126800-pEarleyGate-104 (pUFV2556). These DNA constructs were also co-infiltrated with the nuclear marker *Arabidopsis thaliana* AtWWP1 fused to mCherry (pUFV2224). At approximately 72 h post-agroinfiltration, 1 cm^2^ leaf explants were excised, and YFP and mCherry fluorescence patterns were examined in epidermal cells with a 40x oil immersion objective and a Zeiss LSM510 META inverted laser scanning microscope equipped with argon/helium-neon lasers as excitation sources. For multi-track imaging, YFP was excited with a 488-nm wavelength, and the emission was collected using a 500–530 nm band-pass filter, and mCherry was excited with a 543 nm wavelength, and the emission was collected using a 596–638 nm band-pass filter. The pinhole was typically set to create a 1–1.5-μm optical slice. Post-acquisition image processing was performed using LSM Image Browser 4 software (Carl-Zeiss) and Adobe Photoshop (Adobe Systems).

### GUS activity assays

Leaves from BIP-9::GUS and pCambia::GUS transgenic lines were agroinoculated with Glyma.02G161100-pEarleyGate-104 (pUFV2554), glyma03g27865-pEarleyGate-104 (pUFV2555) or Glyma.19G126800-pEarleyGate-104 (pUFV2556) to express the truncated bZIP proteins. Infiltration with *Agrobacterium tumefaciens* carrying a GFP protein (pUFV1088) expression cassette was used as a control. Untransformed, wild-type leaves were also used as a negative control. At 72 h post-infiltration of the *Agrobacterium* suspension cultures, total leaf protein was extracted, and fluorometric assays of GUS activity were performed essentially as described [61], with methylumbelliferone (MU) as a standard.

### Induction of salt stress and ER stress

For the stress treatments, soybean seeds (cv. Conquista) were germinated in an organic substrate and grown under greenhouse conditions. Fifteen days after germination (V2 stage), roots were washed with water, and the plants were transferred to 200 mmol/L NaCl for 1, 2, 4 and 8 h or 2.5 μg/mL tunicamycin for 4 h for ER stress induction. After all of the treatments, the plant materials were harvested, immediately frozen in liquid N_2_ and stored at −80 °C until use. Each stress treatment and RNA extraction were replicated in three independent samples.

### Isolation of total RNA from soybean leaves and synthesis of cDNA

Total RNA was extracted from frozen leaves with TRIzol (Invitrogen), according to the manufacturer’s instructions. RNA quality and integrity were monitored by electrophoresis on denaturing 1.2 % (w/v) agarose gels stained with 0.1 μg/mL ethidium bromide. First-strand cDNA was synthesized from 3 μg RNase-free DNase I-treated total RNA using oligo-dT primers (18) and M-MLV Reverse Transcriptase (Life Technologies), according to the manufacturer’s instructions.

### Quantitative RT-PCR

The real-time PCR procedures, including the pilot tests, validations, and experiments, were performed according to the information supplied by the Life Technologies manual. Real-time RT-PCR assays were performed with an ABI 7500 instrument (Life Technologies) using SYBR Green PCR Master Mix (Life Technologies) and gene-specific primers (Additional file 16). The conditions for the amplification reactions were as follows: 10 min at 95 °C, followed by 40 cycles at 94 °C for 15 s and 60 °C for 1 min. Absolute gene expression was quantified using the comparative Ct (2^–ΔCt^) method. Expression of soybean genes was normalized to that of an RNA helicase endogenous control gene, and expression of *N. tabacum* genes was normalized to that of actin.

### Analysis of stress-induced splicing of GmbZIP60 mRNA

The presence of hairpin-like structures as potential IRE substrates in the sequences of soybean bZIP60 homologs was examined using RNA folding software in Mfold web server version 2.3 (http://mfold.rna.albany.edu/), with the default parameters. This *in silico* analysis revealed a possible functional spliced bZIP60 transcript derived from the Glyma02g19754 sequence using two sets of primers (Additional files 7 and 15) that were capable of discriminating between spliced and unspliced bZIP60 transcripts in RT-PCR assays. The set of primers glyma02g19754Fwd and glyma02g19754spdR was used for identification of the spliced bZIP60 transcript, whereas the set of primers glyma02g19754Fwd and glyma02g19754unsp amplified the unspliced bZIP60 transcript from cDNA prepared from tunicamycin-treated and untreated total leaf RNA, respectively. Approximately 1 μl of cDNA was used to PCR-amplify the spliced/unspliced transcripts using the indicated pair of primers at a concentration of 0.4 μM each, with 0.2 mM dNTPs, 5 μl of 10X High Fidelity Platinum Taq buffer (Life Technologies) and 0.2 U of High Fidelity Platinum Taq (Life Technologies) in a final volume of 50 μl. The amplification reaction was conducted with an initial denaturation step at 94 °C for 3 min, followed by 28 cycles at 94 °C for 45 s, 55 °C for 45 s, and 68 °C for 45 s and a final extension at 68 °C for 10 min. The amplification products were examined with a silver-stained 15 % (w/v) polyacrylamide gel.

### Statistical analyses

All statistical analyses were performed using R software (http://cran.r-project.org) with the ExpDest package [62]. Statistical analysis of GUS activity data was performed using two-way ANOVA (2x6 factorial design, with a completely randomized design and three repetitions) followed by the Scott-Knott test at a p < 0.05. For the qRT-PCR data, the means were compared using confidence intervals generated by the *t* test at a p ≤ 0.05.

## Additional files

Additional file 1:
**A list of known/predicted orthologous genes involved in the** ***Glycine max*** **and** ***Arabidopsis thaliana*** **UPR pathways.** (XLS 152 kb) Additional file 2:
**Phylogenetic tree based on IRE-like sequences from Arabidopsis, soybean and rice.** The unrooted phylogenetic tree was constructed using the maximum likelihood method with 10,000 bootstrap replications and the Jones-Talor-Thornton (JTT) amino acid substitution model with MEGA v.6 software. The numbers shown at the nodes indicate the percentage bootstrap scores. (TIFF 297 kb) Additional file 3:
**Phylogenetic tree based on membrane-tethered bZIP-like sequences from Arabidopsis, soybean and rice.** The unrooted phylogenetic tree was constructed using the maximum likelihood method with 10,000 bootstrap replications and the Jones-Talor-Thornton (JTT) amino acid substitution model using MEGA v.6 software. The numbers shown at the nodes indicate the percentage bootstrap scores. (TIFF 583 kb) Additional file 4:
**Illustrative scheme of the predicted bZIP38 (A), bZIP37 (B) and bZIP68 (C) primary structures.** The numbers above the figure indicate the amino acid positions in the predicted protein, and the numbers in parentheses indicate the corresponding nucleotide positions in the cDNA sequence. The bZIP domain is denoted in blue, TM is the putative transmembrane segment, S1P is the position of a canonical site for site-1 protease, and NLS indicates the position of a nuclear localization signal. (TIFF 133 kb) Additional file 5:
**Sequence alignments of bZIP17/28-like sequences from Arabidopsis and soybean.** The sequence alignments of the indicated genes were obtained with CLUSTAL-W program. The bZIP domain, the transmembrane segment and a canonical S1P cleavage site are marked by open boxes. (TIFF 3439 kb) Additional file 6:
**Predicted structure of GmbZIP68 mRNA.** The form of Glyma02g19754 mRNA folded by Mfold with the lowest free energy of ∆*G = −*191.80 (initially −187.80). (TIFF 1485 kb) Additional file 7:
**Partial nucleotide and amino acid sequences derived from unspliced and spliced GmbZIP68 mRNAs.** The arrows indicate the putative splicing sites in the unspliced mRNA and the ligation site in the spliced mRNA. The predicted nuclear localization signals (NLSs) are indicated by the amino acid sequences in orange. The predicted transmembrane segment is underlined. The amino acid sequence in red, derived from the spliced mRNA, shows the translational frameshift that resulted in a predicted amino acid sequence that was distinct from that of the unspliced mRNA. The nucleotide sequence in green corresponds to the forward primer, whereas the light blue sequence is complementary to the reverse unspliced primer, and the dark blue sequence is complementary to the reverse spliced primer used in splicing assay. (TIFF 625 kb) Additional file 8:
**Unconventional splicing of GmbZIP68 mRNA A.** Electrophoretic patterns of RT-PCR products of ER stress-induced spliced GmbZIP68 mRNA on 1 % agarose gels. Lanes 1 and 2 show the RT-PCR products generated using total RNA from untreated soybean seedlings with unspliced GmbZIP68 mRNA-specific primers (U, lane 1) and spliced mRNA-specific primers (S, lane 2). The RT-PCR products generated from RNA of soybean seedlings treated with tunicamycin for 8 h and 24 h are shown in lanes 3–6 using unspliced GmbZIP68 mRNA-specific primers (U, lanes 4 and 6) and spliced mRNA-specific primers (S, lanes 3 and 5). B. ER stress-induced unconventional splicing of AtbZIP60 mRNA. Total RNA from Arabidopsis seedlings treated for 6 h with tunicamycin was used as a template for RT-PCR performed with spliced bZIP60 mRNA-specific primers in combination with unspliced bZIP60 mRNA-specific primers. (TIFF 272 kb) Additional file 9:
**Phylogenetic tree based on HSP70-like sequences from** ***Glycine max*** **and** ***Arabidopsis thaliana*** **.** The unrooted phylogenetic tree was constructed using the maximum likelihood method with 10,000 bootstrap replications and the Jones-Talor-Thornton (JTT) amino acid substitution model with MEGA v.6 software. The numbers shown at the nodes indicate the percentage bootstrap scores. (TIFF 1528 kb) Additional file 10:
**Phylogenetic tree based on PDI-like sequences from** ***Glycine max*** **and** ***Arabidopsis thaliana*** **.** The unrooted phylogenetic tree was constructed using the maximum likelihood method with 10,000 bootstrap replications and the Jones-Talor-Thornton (JTT) amino acid substitution model with MEGA v.6 software. The numbers shown at the nodes indicate the percentage bootstrap scores. (TIFF 5306 kb) Additional file 11:
**A list of predicted/known orthologous genes involved in** ***Glycine max*** **and** ***Arabidopsis thaliana*** **plant-specific ER stress-mediated cell death pathways.** (XLS 89 kb) Additional file 12:
**Phylogenetic tree based on DCD/NPP-like sequences from** ***Glycine max*** **and** ***Arabidopsis thaliana*** **.** The unrooted phylogenetic tree was constructed using the maximum likelihood method with 10,000 bootstrap replications and the Jones-Talor-Thornton (JTT) amino acid substitution model with MEGA v.6 software. The numbers shown at the nodes indicate the percentage bootstrap scores. (TIFF 439 kb) Additional file 13:
**Phylogenetic tree based on NAC-like sequences from** ***Glycine max*** **and** ***Arabidopsis thaliana.*** The unrooted phylogenetic tree was constructed using the maximum likelihood method with 10,000 bootstrap replications and the Jones-Talor-Thornton (JTT) amino acid substitution model with MEGA v.6 software. The numbers shown at the nodes indicate the percentage bootstrap scores. (TIFF 1018 kb) Additional file 14:
**Phylogenetic tree based on VPE-like sequences from** ***Glycine max*** **and** ***Arabidopsis thaliana.*** The unrooted phylogenetic tree was constructed using the maximum likelihood method with 10,000 bootstrap replications and the Jones-Talor-Thornton (JTT) amino acid substitution model with MEGA v.6 software. The numbers shown at the nodes indicate the percentage bootstrap scores. (TIFF 604 kb) Additional file 15:
**Primers used for PCR.** (DOCX 14 kb) Additional file 16:
**List of gene-specific primers used for qRT-PCR.** (DOCX 73 kb)

## Abbreviations

ER: endoplasmic reticulum
UPR: unfolded protein response
ERAD: endoplasmic reticulum protein degradation
ERSE: ER stress cis-acting element
UPRE: unfolded protein responsive element
IRE1: inositol-requiring transmembrane kinase and endonuclease 1α
BiP: binding protein
PDI: protein disulfide isomerase
CNX: calnexin
CRT: calreticulin
TF: transcription factor
PCD: programmed cell death
NRP/DCD: developmental cell death (DCD) domain-containing N-rich protein (NRP)
GmERD15: *Glycine max* early responsive to dehydration 15
VPE: vacuolar processing enzyme
NAC: no apical meristem (NAM), Arabidopsis ATAF1/2, and cup-shaped cotyledon (CUC)
