# Supplementary figures and images for: Comprehensive analysis of the endoplasmic reticulum stress response in the soybean genome: conserved and plant-specific features
Source: BMC Genomics. 2015 Oct 14;16:783. doi: 10.1186/s12864-015-1952-z (PMC4606518; doi:10.1186/s12864-015-1952-z)

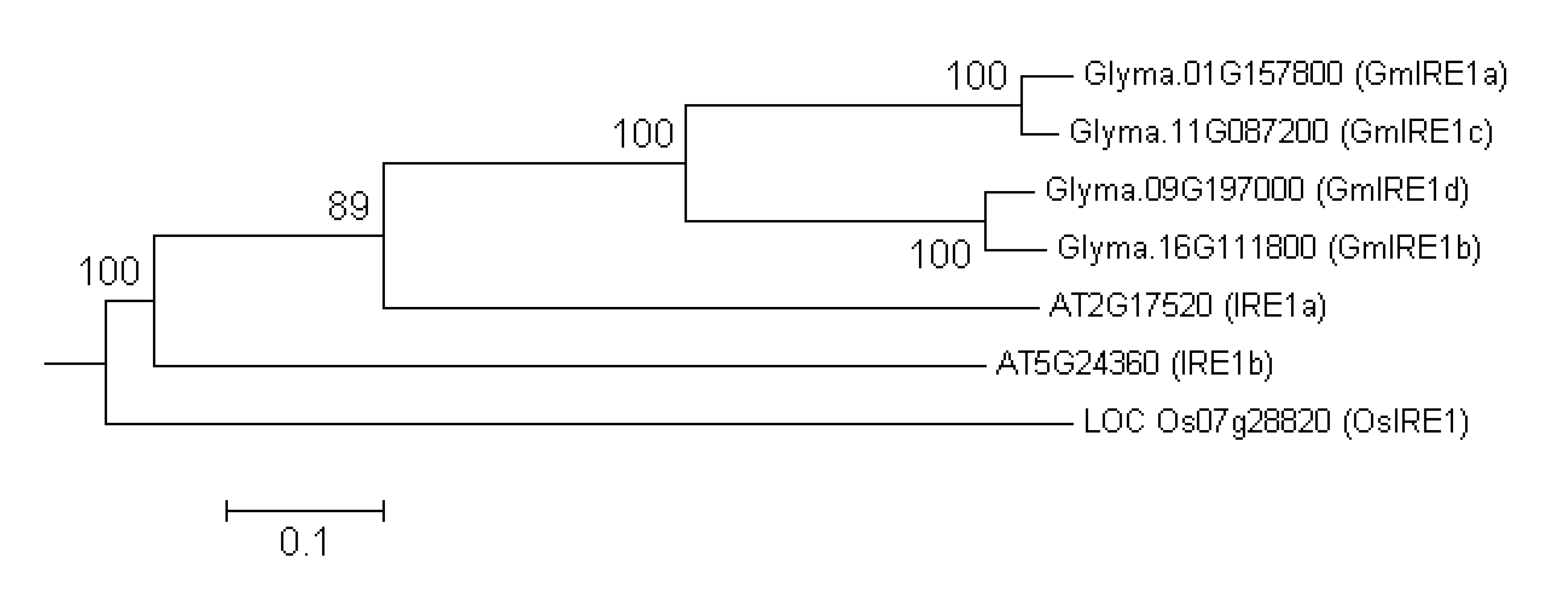

Supplement: Additional file 2: — Phylogenetic tree based on IRE-like sequences from Arabidopsis, soybean and rice. The unrooted phylogenetic tree was constructed using the maximum likelihood method with 10,000 bootstrap replications and the Jones-Talor-Thornton (JTT) amino acid substitution model with MEGA v.6 software. The numbers shown at the nodes indicate the percentage bootstrap scores. (TIFF 297 kb) [file 12864_2015_1952_MOESM2_ESM.tif]

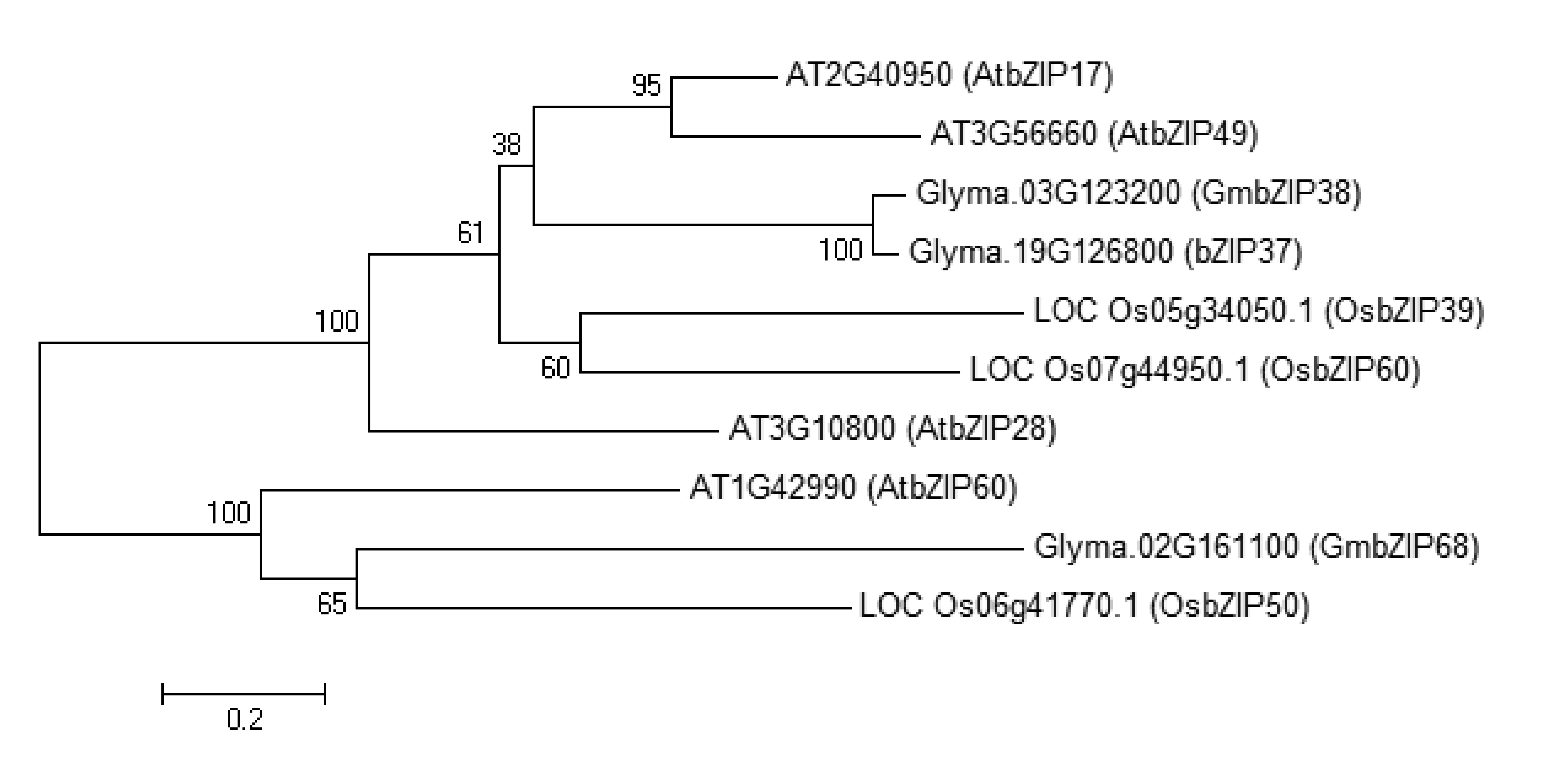

Supplement: Additional file 3: — Phylogenetic tree based on membrane-tethered bZIP-like sequences from Arabidopsis, soybean and rice. The unrooted phylogenetic tree was constructed using the maximum likelihood method with 10,000 bootstrap replications and the Jones-Talor-Thornton (JTT) amino acid substitution model using MEGA v.6 software. The numbers shown at the nodes indicate the percentage bootstrap scores. (TIFF 583 kb) [file 12864_2015_1952_MOESM3_ESM.tif]

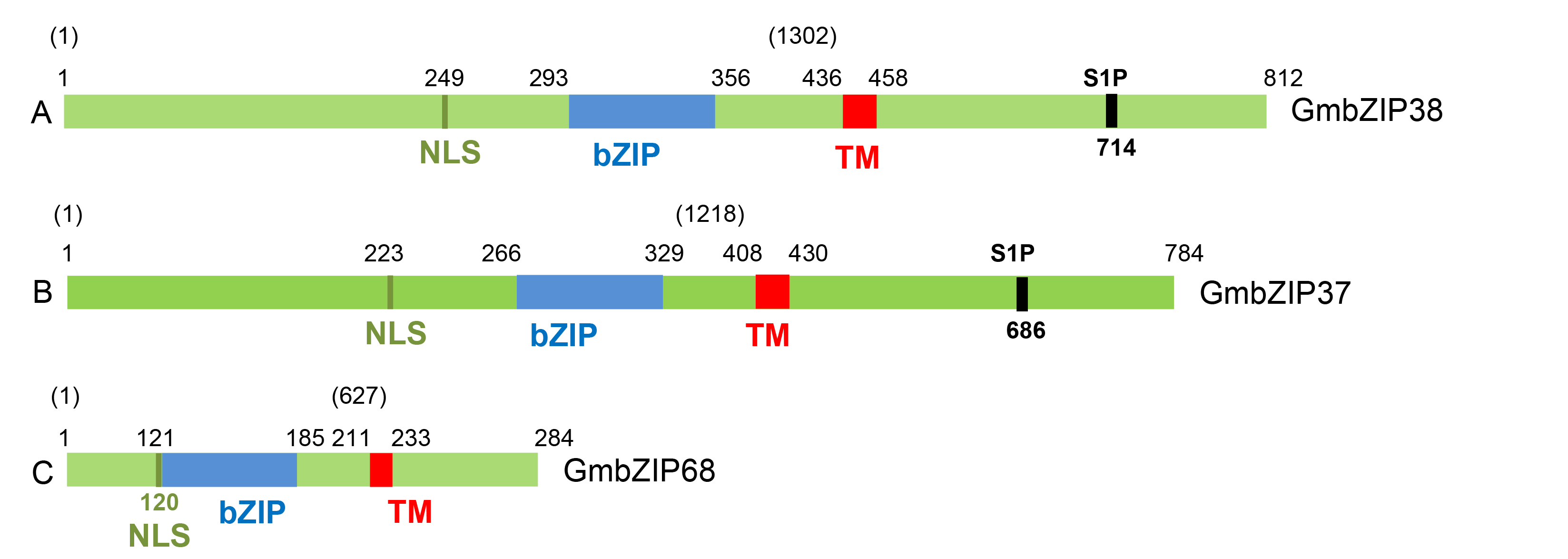

Supplement: Additional file 4: — Illustrative scheme of the predicted bZIP38 (A), bZIP37 (B) and bZIP68 (C) primary structures. The numbers above the figure indicate the amino acid positions in the predicted protein, and the numbers in parentheses indicate the corresponding nucleotide positions in the cDNA sequence. The bZIP domain is denoted in blue, TM is the putative transmembrane segment, S1P is the position of a canonical site for site-1 protease, and NLS indicates the position of a nuclear localization signal. (TIFF 133 kb) [file 12864_2015_1952_MOESM4_ESM.tif]

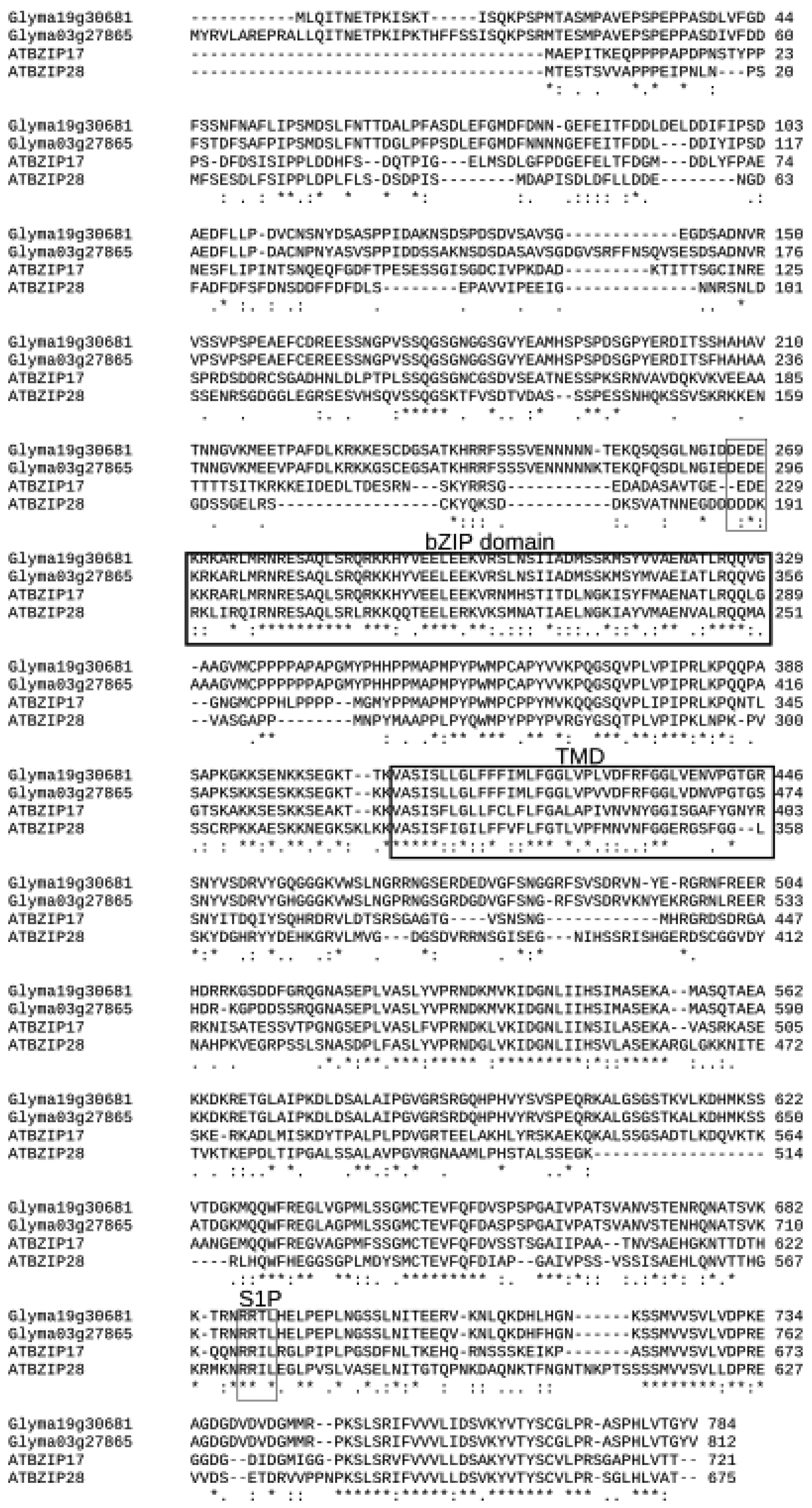

Supplement: Additional file 5: — Sequence alignments of bZIP17/28-like sequences from Arabidopsis and soybean. The sequence alignments of the indicated genes were obtained with CLUSTAL-W program. The bZIP domain, the transmembrane segment and a canonical S1P cleavage site are marked by open boxes. (TIFF 3439 kb) [file 12864_2015_1952_MOESM5_ESM.tif]

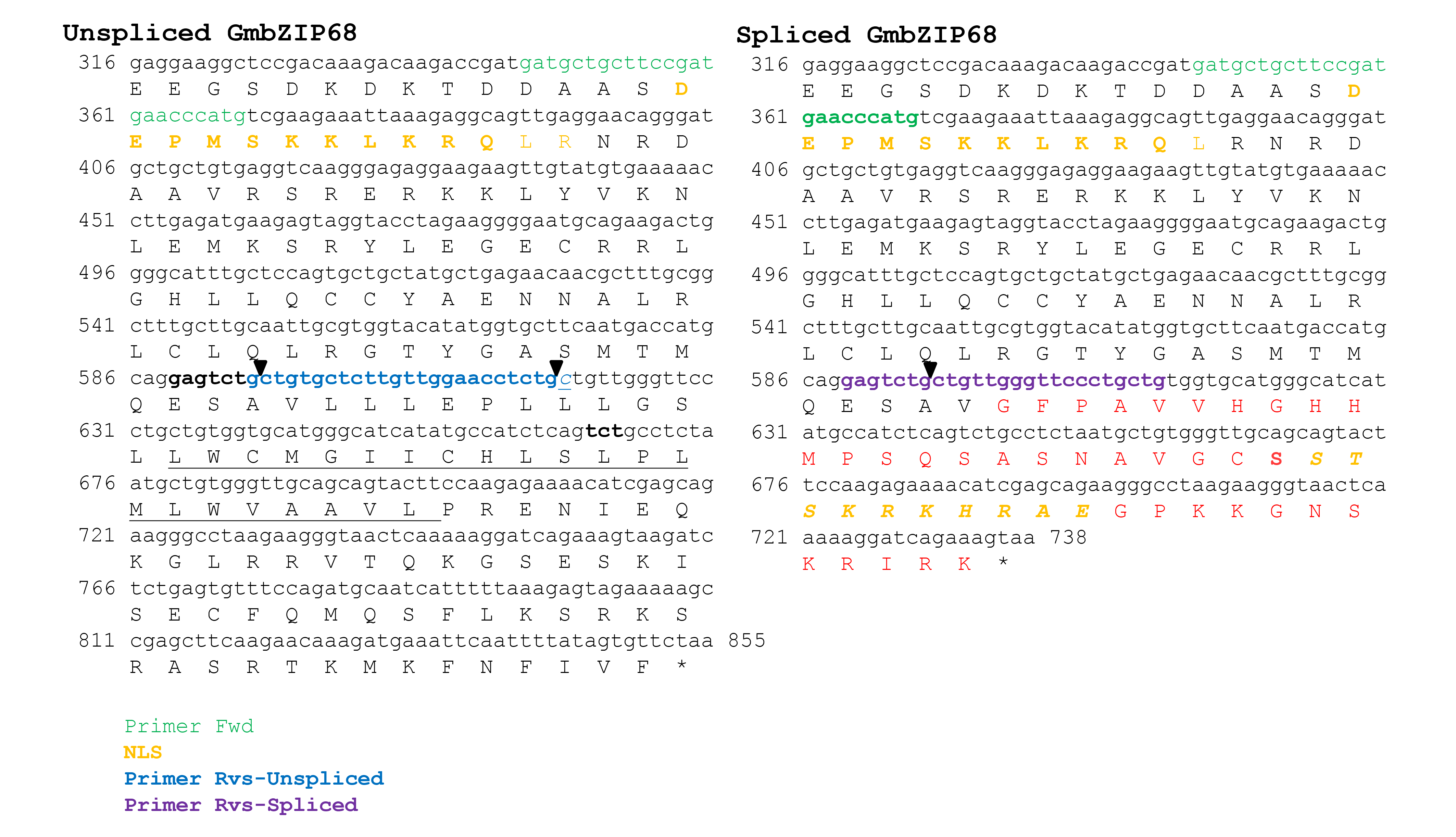

Supplement: Additional file 7: — Partial nucleotide and amino acid sequences derived from unspliced and spliced GmbZIP68 mRNAs. The arrows indicate the putative splicing sites in the unspliced mRNA and the ligation site in the spliced mRNA. The predicted nuclear localization signals (NLSs) are indicated by the amino acid sequences in orange. The predicted transmembrane segment is underlined. The amino acid sequence in red, derived from the spliced mRNA, shows the translational frameshift that resulted in a predicted amino acid sequence that was distinct from that of the unspliced mRNA. The nucleotide sequence in green corresponds to the forward primer, whereas the light blue sequence is complementary to the reverse unspliced primer, and the dark blue sequence is complementary to the reverse spliced primer used in splicing assay. (TIFF 625 kb) [file 12864_2015_1952_MOESM7_ESM.tif]

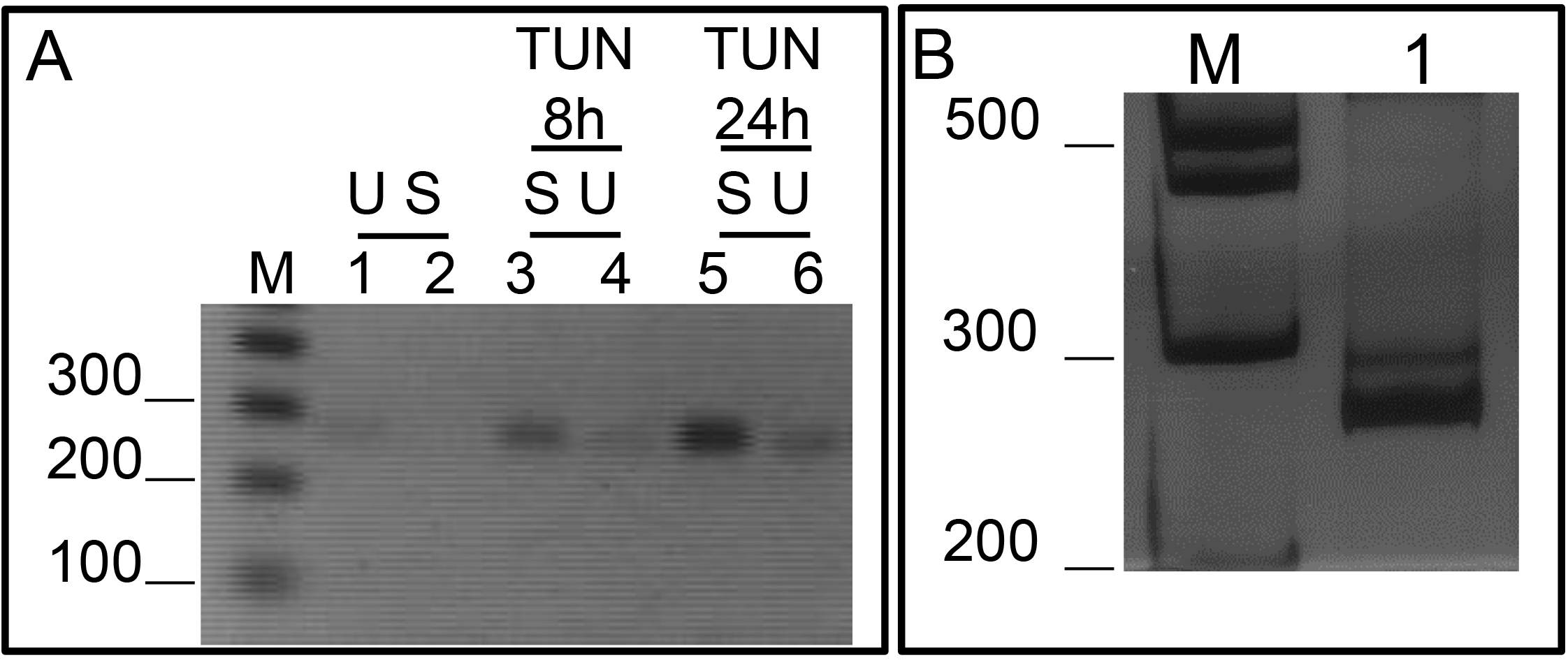

Supplement: Additional file 8: — Unconventional splicing of GmbZIP68 mRNA A. Electrophoretic patterns of RT-PCR products of ER stress-induced spliced GmbZIP68 mRNA on 1 % agarose gels. Lanes 1 and 2 show the RT-PCR products generated using total RNA from untreated soybean seedlings with unspliced GmbZIP68 mRNA-specific primers (U, lane 1) and spliced mRNA-specific primers (S, lane 2). The RT-PCR products generated from RNA of soybean seedlings treated with tunicamycin for 8 h and 24 h are shown in lanes 3–6 using unspliced GmbZIP68 mRNA-specific primers (U, lanes 4 and 6) and spliced mRNA-specific primers (S, lanes 3 and 5). B. ER stress-induced unconventional splicing of AtbZIP60 mRNA. Total RNA from Arabidopsis seedlings treated for 6 h with tunicamycin was used as a template for RT-PCR performed with spliced bZIP60 mRNA-specific primers in combination with unspliced bZIP60 mRNA-specific primers. (TIFF 272 kb) [file 12864_2015_1952_MOESM8_ESM.tif]

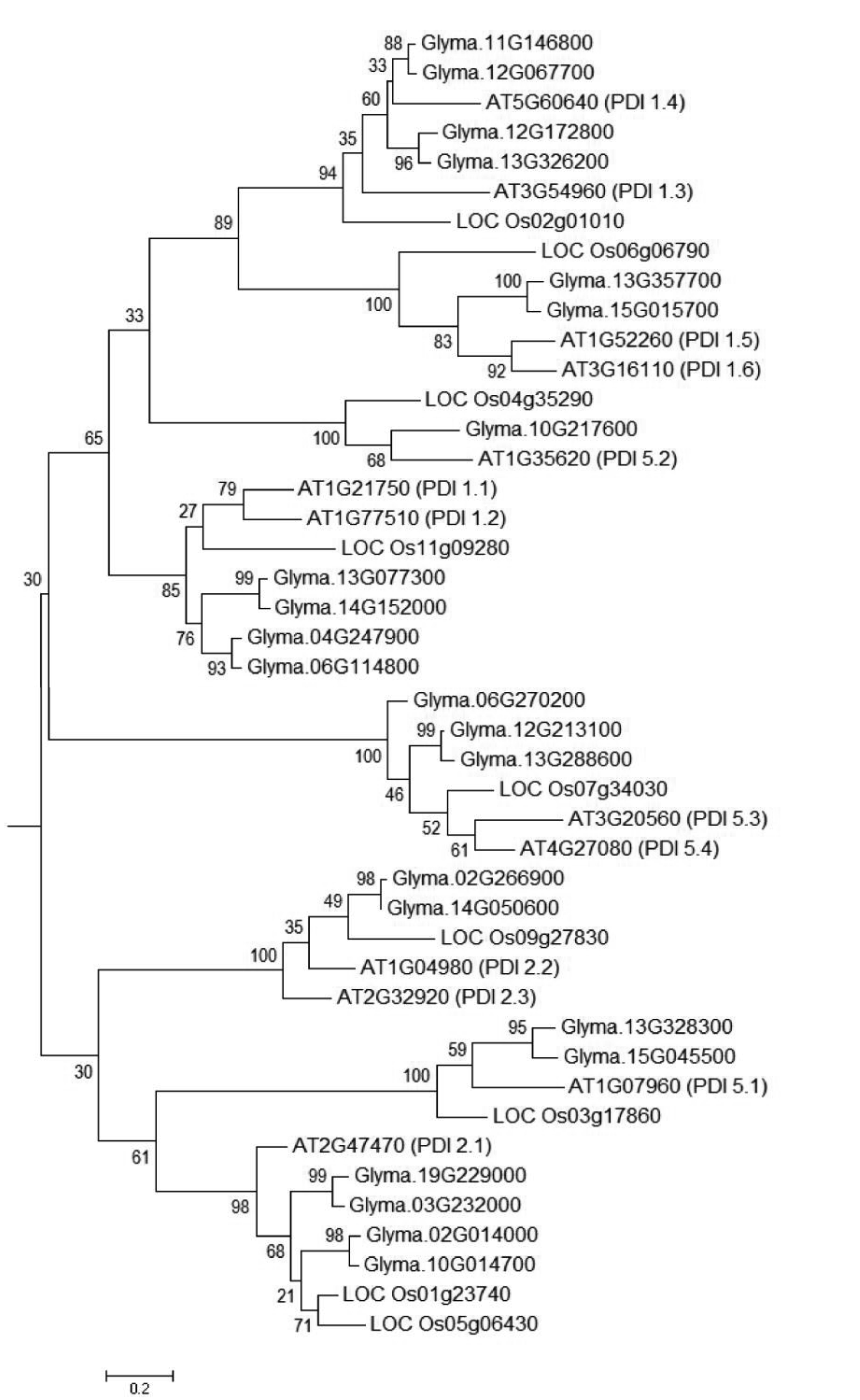

Supplement: Additional file 10: — Phylogenetic tree based on PDI-like sequences from Glycine max and Arabidopsis thaliana . The unrooted phylogenetic tree was constructed using the maximum likelihood method with 10,000 bootstrap replications and the Jones-Talor-Thornton (JTT) amino acid substitution model with MEGA v.6 software. The numbers shown at the nodes indicate the percentage bootstrap scores. (TIFF 5306 kb) [file 12864_2015_1952_MOESM10_ESM.tif]

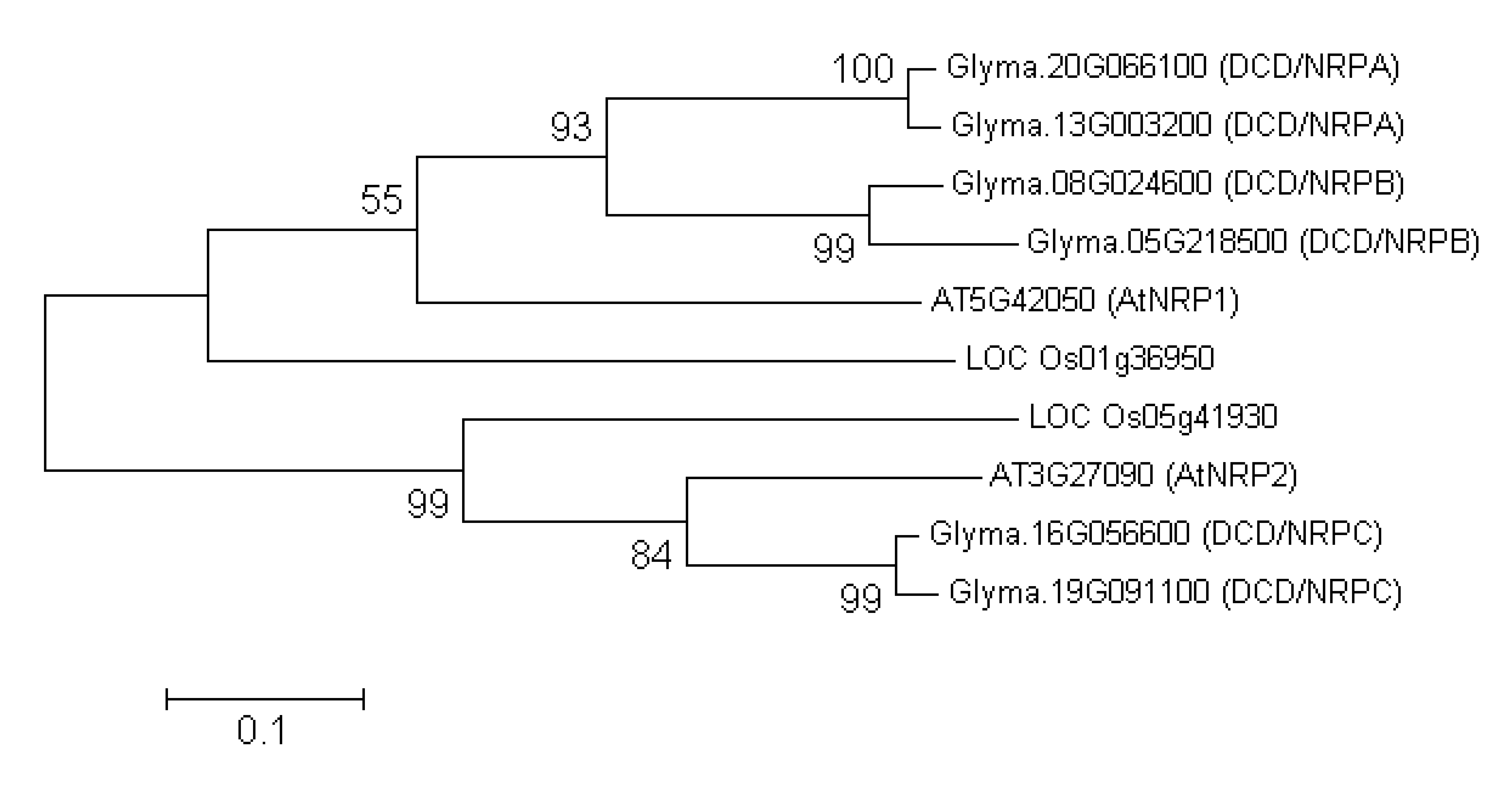

Supplement: Additional file 12: — Phylogenetic tree based on DCD/NPP-like sequences from Glycine max and Arabidopsis thaliana . The unrooted phylogenetic tree was constructed using the maximum likelihood method with 10,000 bootstrap replications and the Jones-Talor-Thornton (JTT) amino acid substitution model with MEGA v.6 software. The numbers shown at the nodes indicate the percentage bootstrap scores. (TIFF 439 kb) [file 12864_2015_1952_MOESM12_ESM.tif]

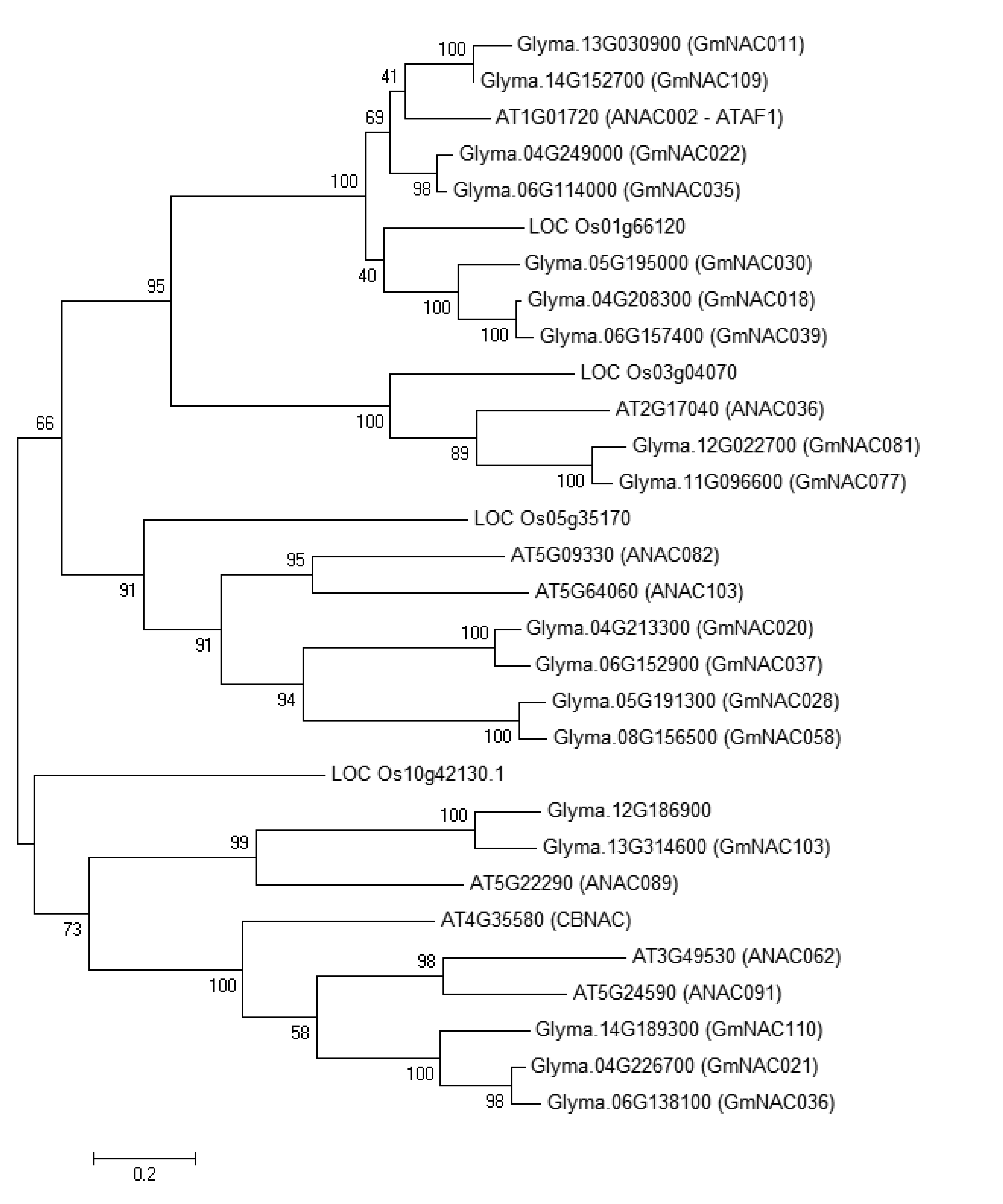

Supplement: Additional file 13: — Phylogenetic tree based on NAC-like sequences from Glycine max and Arabidopsis thaliana. The unrooted phylogenetic tree was constructed using the maximum likelihood method with 10,000 bootstrap replications and the Jones-Talor-Thornton (JTT) amino acid substitution model with MEGA v.6 software. The numbers shown at the nodes indicate the percentage bootstrap scores. (TIFF 1018 kb) [file 12864_2015_1952_MOESM13_ESM.tif]
