# Supplementary material for: Comprehensive analysis of the endoplasmic reticulum stress response in the soybean genome: conserved and plant-specific features
Source: BMC Genomics. 2015 Oct 14;16:783. doi: 10.1186/s12864-015-1952-z (PMC4606518; doi:10.1186/s12864-015-1952-z)
Supplement: Additional file 15: — Primers used for PCR. (DOCX 14 kb) [file 12864_2015_1952_MOESM15_ESM.docx]

**Additional file 15. Primers used for PCR**

| Targeted sequence | | Name | | Primer sequence (5’- 3’) | |
| --- | --- | --- | --- | --- | --- |
| Glyma02g19754 | Glyma02g19754GW-Fw | | AAAAAGCAGGCTTCACAATGGACGAATTAGAAGAAAC | |  |
| Glyma02g19754 | Glyma02g19754GW-Rv | | AGAAAGCTGGGTCACCCAACAGCAGAGGTTCCAAC | |  |
| Glyma19g30681 | Glyma19g30681GW-Fw | | AAAAAGCAGGCTTCACAATGCTTAAAATAACTAACG | |  |
| Glyma19g30681 | Glyma19g30681GW-Rv | | AGAAAGCTGGGTCCGTAGTTTTCCCCTCACTCTT | |  |
| Glyma03g27865 | Glyma03g27865Gw2-Fw | | AAAAAGCAGGCTTCACAATGTACAGGGTTTTAGCGCG | |  |
| Glyma03g27865 | Glyma03g27865Gw2-Rv | | AGAAAGCTGGGTCTTCTTA GTT TTC CCC TCA CTC TTC | |  |
| attB1 | 2942 | | GGGGACAAGTTTGTACAAAAAAGCAGGCT | |  |
| attB2 | 2943 | | GGGGACCACTTTGTACAAGAAAGCTGGGT | |  |
| pDONR 207 | 3397 (Fwd) | | TCGCGTTAACGCTAGCATGGATC | |  |
| pDONR 207 | 3398 (Rvs) | | TGTAACATCAGAGATTTTGAGACAC | |  |
| 35S | MC36 (Fwd) | | TCCTTCGCAAGACCCTTCCTC | |  |
| Glyma02g19754 | glyma02g19754Fwd | | GATGCTGCTTCCGATGAACCCATG | |  |
| Glyma02g19754 | glyma02g19754unspR | | GCAGAGGTTCCAACAAGAGCACAG | |  |
| Glyma02g19754 | glyma02g19754spdR | | CAGCAGGGAACCCAACAGCAGACTC | |  |
