# Supplementary material for: Comprehensive analysis of the endoplasmic reticulum stress response in the soybean genome: conserved and plant-specific features
Source: BMC Genomics. 2015 Oct 14;16:783. doi: 10.1186/s12864-015-1952-z (PMC4606518; doi:10.1186/s12864-015-1952-z)
Supplement: Additional file 16: — List of gene-specific primers used for qRT-PCR. (DOCX 73 kb) [file 12864_2015_1952_MOESM16_ESM.docx]

**Additional file 16. List of gene-specific primers for qRT-PCR**

| Primer | Sequence (5’ – 3’) | Gene |
| --- | --- | --- |
| RT-Glyma19g30681FWD | GGTGCTGCTGGTGTCATGTG | Glyma19g30681 |
| RT-Glyma19g30681-RVS | ATAGGTGCCATTGGAGGATGAT | Glyma19g30681 |
| RT-Glyma03g27865-FWD | TCGACGACCTCGATGACATC | Glyma03g27865 |
| RT-Glyma03g27865-RVS | GCGAAACGGAGGCGTAATTA | Glyma03g27865 |
| RT-Glyma02g19754-FWD | GCTTCCGATGAACCCATGTC | Glyma02g19754 |
| RT-Glyma02g19754-RVS | TTCCTCTCCCTTGACCTCACA | Glyma02g19754 |
| HelicFw | TAACCCTAGCCCCTTCGCCT | HELIC |
| HelicRv | GCCTTGTCGTCTTCCTCCTCG | HELIC |
| BIPDFW | ATCTGGAGGAGCCCTAGGCGGTGG | BIPD |
| BIPDRV | CTTGAAGAAGCTTCGTCGTAAAACTAAG | BIPD |
| SMPFW | GCCGAACTGAGGAAAAGACGAACC | SMP |
| SMPRV | CTTGGGCTGTTTGTTGGGTCTTC | SMP |
| CALNFW | TGATGGGGAGGAGAAGAAAAAGGC | CNX |
| CALNRV | CACTTGGGTTTGGGATCTTGGCTC | CNX |
| Nac2 Fw | GGGTGCTTTGCCGTATTTACAA | GmNAC35 |
| Nac2 Rv | CTCCTCCGCTTTTCAGAATCTC | GmNAC35 |
| ActinFwd |  | NbActin |
| ActinRvs |  | NbActin |
